# Multimodal soft tissue markers for bridging high-resolution diagnostic imaging with therapeutic intervention

**DOI:** 10.1126/sciadv.abb5353

**Published:** 2020-08-19

**Authors:** Anders E. Hansen, Jonas R. Henriksen, Rasmus I. Jølck, Frederikke P. Fliedner, Linda M. Bruun, Jonas Scherman, Andreas I. Jensen, Per. Munck af Rosenschöld, Lilah Moorman, Sorel Kurbegovic, Steen R. de Blanck, Klaus R. Larsen, Paul F. Clementsen, Anders N. Christensen, Mads H. Clausen, Wenbo Wang, Paul Kempen, Merete Christensen, Niels-Erik Viby, Gitte Persson, Rasmus Larsen, Knut Conradsen, Fintan J. McEvoy, Andreas Kjaer, Thomas Eriksen, Thomas L. Andresen

**Affiliations:** 1 DTU Health Technology, Section for Biotherapeutic Engineering and Drug Targeting, Center for Nanomedicine and Theranostics, Technical University of Denmark, Kgs. Lyngby DK-2800, Denmark.; 2 Dept. of Clinical Physiology, Nuclear Medicine & PET and Cluster for Molecular Imaging, Dept. of Biomedical Sciences, Copenhagen University Hospital (Rigshospitalet) and University of Copenhagen, Copenhagen, DK-2200, Denmark.; 3 Radiation Physics, Department of Hematology, Oncology and Radiation Physics, Skåne University Hospital, Lund SE-222 42, Sweden.; 4 DTU Health Technology, The Hevesy Laboratory, Technical University of Denmark, Roskilde DK-4000, Denmark.; 5 Department of Veterinary Clinical and Animal Sciences, University of Copenhagen, Frederiksberg DK-1870, Denmark.; 6 Department of Oncology, Copenhagen University Hospital (Rigshospitalet), Copenhagen DK-2100, Denmark.; 7 Department of Respiratory Medicine, Copenhagen University Hospital (Bispebjerg and Frederiksberg Hospital), Copenhagen DK-2400, Denmark.; 8 Copenhagen Academy for Medical Education and Simulation (CAMES), Department of Internal Medicine, Zealand University Hospital, Roskilde, Denmark.; 9 Department of Clinical Medicine, University of Copenhagen, Copenhagen, DK-2100, Denmark.; 10 DTU Compute, Section for Applied Mathematics and Computer Science, Technical University of Denmark, Kgs. Lyngby DK-2800, Denmark.; 11 Department of Cardiothoracic Surgery, Copenhagen University Hospital (Rigshospitalet), Copenhagen DK-2100, Denmark.; 12 Department of Oncology, Herlev-Gentofte Hospital, Department of Clinical Medicine, Faculty of Health Science, University of Copenhagen, Copenhagen DK-2200, Denmark.

## Abstract

Diagnostic imaging often outperforms the surgeon’s ability to identify small structures during therapeutic procedures. Smart soft tissue markers that translate the sensitivity of diagnostic imaging into optimal therapeutic intervention are therefore highly warranted. This paper presents a unique adaptable liquid soft tissue marker system based on functionalized carbohydrates (Carbo-gel). The liquid state of these markers allows for high-precision placement under image guidance using thin needles. Based on step-by-step modifications, the image features and mechanical properties of markers can be optimized to bridge diagnostic imaging and specific therapeutic interventions. The performance of Carbo-gel is demonstrated for markers that (i) have radiographic, magnetic resonance, and ultrasound visibility; (ii) are palpable and visible; and (iii) are localizable by near-infrared fluorescence and radio guidance. The study demonstrates encouraging proof of concept for the liquid marker system as a well-tolerated multimodal imaging marker that can improve image-guided radiotherapy and surgical interventions, including robotic surgery.

## Teaser

The liquid-based Carbo-gel technology enables real-time multimodal image guidance for improved therapeutic precision.

## INTRODUCTION

Diagnostic imaging has undergone tremendous development in terms of precision and accuracy, and solid malignancies are identified at increasingly smaller sizes and at earlier stages. This has revolutionized the ability to accurately plan radiation therapy and surgical interventions, resulting in improved patient outcomes (*1*, *2*). However, using diagnostic imaging of such resolution for therapeutic intervention has proven difficult and has identified unmet needs for smart fiducial markers that allow surgeons to translate image findings to therapy.

Image-guided radiation therapy (IGRT) has improved the precision and enabled the reduction in safety margins, which has lowered treatment-associated toxicities (*3*). IGRT is inherently dependent on accurate localization of the cancerous tissue from diagnostic imaging through the radiotherapy course, e.g., via three dimensional cone-beam computed tomography (CBCT), which is a nontrivial procedure for a number of locations and tissues. As an example of this, suboptimal CBCT contrast is encountered when cancerous and surrounding tissues have too similar radiographic attenuation, which may require the use of large treatment safety margins (*4*). Solid radiopaque soft tissue markers provide a possible solution to this problem. However, their application is challenging for a number of indications in terms of patient discomfort, placement and marker migration, proton therapy dose perturbation, and distortion of the planning images (*5*, *6*). These challenges are highlighted in study reports of patients with lung cancer where percutaneous insertion of markers was associated with pneumothorax and bronchoscopic procedures with dislodging and migration (*7*–*10*). For IGRT in patients with prostate cancer, transrectal ultrasound-guided insertion of metallic markers using large-gauge needles has been associated with urination problems and symptomatic infections despite antibiotic prophylaxis (*11*, *12*). The ideal marker for IGRT should be visible across radiography-based modalities, magnetic resonance imaging (MRI), and ultrasonography (US); be administered using minimally invasive techniques; display positional and geometrical stability; and maintain imaging properties throughout the course of therapy.

Flexible markers with multimodal imaging properties are not only of interest for radiotherapy guidance. Surgical oncologists are often challenged in localization of diseased tissue in patients diagnosed with small lesions during, e.g., lung and breast cancer screening programs (*12*–*14*). Video-assisted thoracic surgery (VATS) is often the method of choice to resect small pulmonary lesions (*15*). However, intraoperative localization of small lesions can be challenging, as these are often nonvisible and nonpalpable (*16*–*18*). Modern mammography identifies lesions at increasingly smaller sizes, which are challenging for surgeons to accurately locate and excise. In an attempt to improve surgical outcome, wire-guided localization (WGL) and radio-guided occult lesion localization (ROLL) have been applied. The WGL technique has been associated with complications, including wire migration and transection, interference with the surgical procedure, and patient discomfort (*14*, *19*, *20*). The ROLL technology, where technetium-99m–labeled human serum albumin colloids are injected directly into tumors, and the use of colored or near-infrared (NIR) fluorescent dyes were therefore developed as possible alternatives (*14*, *21*). These technologies suffer from rapid dispersion of the marker material, resulting in inefficient surgical guidance. Furthermore, technetium-99m has a short half-life, and verification of the position of injection is not possible on mammography. To circumvent the dispersion of radioactivity, the use of low-activity iodine-125 (half-life, ~59 days) metallic seeds was investigated for surgical guidance, but the insertion of seeds requires large-gauge needles, and patient discomfort has been reported (*14*, *20*, *22*). The observed benefit of surgical guidance has expanded the use of ROLL techniques to solitary lung tumors and thyroid carcinomas (*23*), and encouraging data have been reported for intraoperative localization of atypical lesions (*24*).

Despite efforts to improve current soft tissue markers, those available for radiation therapy and surgical guidance have shortcomings in terms of practicability and accuracy in translation of diagnostic image findings into therapeutic intervention. To bridge this gap, we have developed a liquid fiducial marker system, Carbo-gel, that allows for visualization across multiple imaging techniques, simple injection via small-gauge needles, and provides accurate and precise guidance of radiotherapy and surgical procedures. Upon injection of Carbo-gel into soft tissue, the liquid marker forms a stable well-defined marker at the site of injection. Injection and clinical performance of Carbo-gel with radiographic contrast have been demonstrated in patients with locally advanced non–small cell lung cancer and esophageal cancer. Encouragingly, none of the included patients developed pneumothorax associated with the marker injection, and negligible migration and stable marker contrast were observed (*25*, *26*). Following this, we expanded the Carbo-gel system to include clinically requested imaging properties. We have evaluated the clinical value of the marker system with a stepwise addition of image and palpatory properties across preclinical translational models. This study presents marker designs for radiotherapy and conventional, image-guided, and robotic surgery to demonstrate (i) radiographic contrast (X-mark) with sequential addition of (ii) soft tissue palpatory and visual properties (XPV-mark), (iii) NIR fluorescence (XPVN-mark), and, last, (iv) radionuclides for radiation-based image guidance (XPVN-[^64^Cu]-mark and XPVN-[^125^I]-mark).

## RESULTS

### Carbo-gel: A liquid carbohydrate-based multimodal marker system

**Fig. 1:**
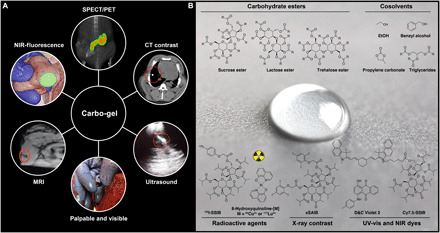
Applications for the Carbo-gel marker system and chemical structures constituting the Carbo-gel liquid marker system. (**A**) The liquid soft tissue marker may be administered with high precision to virtually all locations accessible by small-gauge needles. The markers were developed for guiding radiotherapy, as well as conventional, image-guided, and robotic surgery that each benefit from fiducial markers. The Carbo-gel markers are intrinsically visible on US and MRI, and sequential addition of further imaging properties is demonstrated with X-mark that also has radiographic/x-ray contrast, XPV-mark that additionally is visible and palpable in soft tissue, and XPVN-mark that has the additional property of being visible using NIR fluorescence imaging. PET and SPECT imaging and radiosurgery properties were included in XPVN-[^125^I]-mark and XPVN-[^64^Cu]-mark by the incorporation of radionuclides (Photo credit: Jonas R. Henriksen, Technical University of Denmark). (**B**) The unique marker properties such as MRI and US contrast and palpability are obtained by dissolving carbohydrate esters with cosolvents. Additional imaging functionalities are acquired by adding radioactive or x-ray contrast agents or ultraviolet-visible (UV-vis) or NIR dyes. R = Me/Et/iPr for the carbohydrate esters, and R = pentyl, heptyl for the triglycerides (Photo credit: Jonas R. Henriksen, Technical University of Denmark).

Carbo-gel is a liquid marker system intended for use in soft tissues. The liquid properties allow for percutaneous and endoscopic injection using small-gauge needles, with high positional accuracy and precision using image guidance. After injection into soft tissue, the liquid forms positionally stable markers with characteristics optimized for several imaging modalities (Fig. 1A).

Carbo-gel is based on esterified carbohydrates such as sucrose, lactose, or trehalose scaffolds (Fig. 1B) with varying hydrophobicities depending on the acyl substituents (acetate, propionate, or isobutyrate). These carbohydrate esters form homogeneous injectable liquids when dissolved in biocompatible cosolvents such as ethanol (EtOH), benzyl alcohol, propylene carbonate, or liquid triglycerides (Fig. 1B). Synthetic chemical functionalization of the carbohydrate esters and modification of the marker composition allow for inclusion of unique features, for example, computed tomography (CT), US, MRI, NIR fluorescence, colored dyes, single photon emission computed tomography (SPECT), and positron emission tomography (PET) in a step-by-step manner. The physical properties of the marker can be further optimized on the basis of the chosen carbohydrate scaffold to give either highly viscous gel-like markers [sucrose acetate isobutyrate (SAIB)] or amorphous solid markers [lactose octaisobutyrate (LOIB)] for in situ palpation (Fig. 1B and fig. S1, and Supplementary Materials). Once injected into tissue or water, the liquid marker undergoes nonsolvent-induced phase separation, where the cosolvent diffuses into the surrounding soft tissue. The limited water content, rigid texture, and hydrophobicity of Carbo-gel render the markers intrinsically visible on US and as signal void regions on T1- and T2-weighted MRI. The US characteristics enable real-time image guidance during injection (movie S1) (*27*). On the basis of cosolvent polarity, different efflux rates may be achieved, which affect the mechanical and physiochemical properties of the markers in tissues. Generally, the more polar solvents, such as ethanol, diffuse more readily from the markers, whereas less polar solvents, such as benzyl alcohol or triglycerides, are retained to a greater extent. Upon cosolvent efflux, the markers form structures with varying degrees of porosity, as shown by cryo–scanning electron microscopy (fig. S2, A and B).

### X-mark: A liquid radiographic contrast soft tissue marker

The injectability through small-gauge needles makes Carbo-gel highly attractive as an IGRT fiducial marker if sufficient radiographic opacity and positional stability can be achieved. For this purpose, the electron-dense iodinated compound, xSAIB (Fig. 1B), was synthesized as a mixed isobutyrate, triiodophenoxyacetate (TIPA) sucrose ester derivative (Supplementary Materials, fig. S1, synthesis of xSAIB) to provide radiographic contrast with minimal image distortion (*6*). This provides a substantial advantage compared with artifacts introduced on x-ray– and MRI-based images by current metal-based fiducials (*27*). Incorporation of increasing amounts of xSAIB in Carbo-gels (SAIB or LOIB based) provided an excellent CT contrast of 1000 to 9000 Hounsfield units (HU) (Supplementary Materials; fig. S2, C and D). The aromatic organo-bound iodine (TIPA) and high hydrophobicity of xSAIB (log *P* = 15.1) (*28*) render xSAIB weakly soluble in ethanol and insoluble in water, resulting in complete retention of xSAIB with only minimal degradation of the compound observed over 99 days in buffers from pH 5 to 8 (Supplementary Materials; fig. S3, A and B). The performance and applications of xSAIB formulated as X-mark (SAIB:xSAIB:EtOH, 50:30:20) were evaluated and advanced into preclinical testing.

**Fig. 2:**
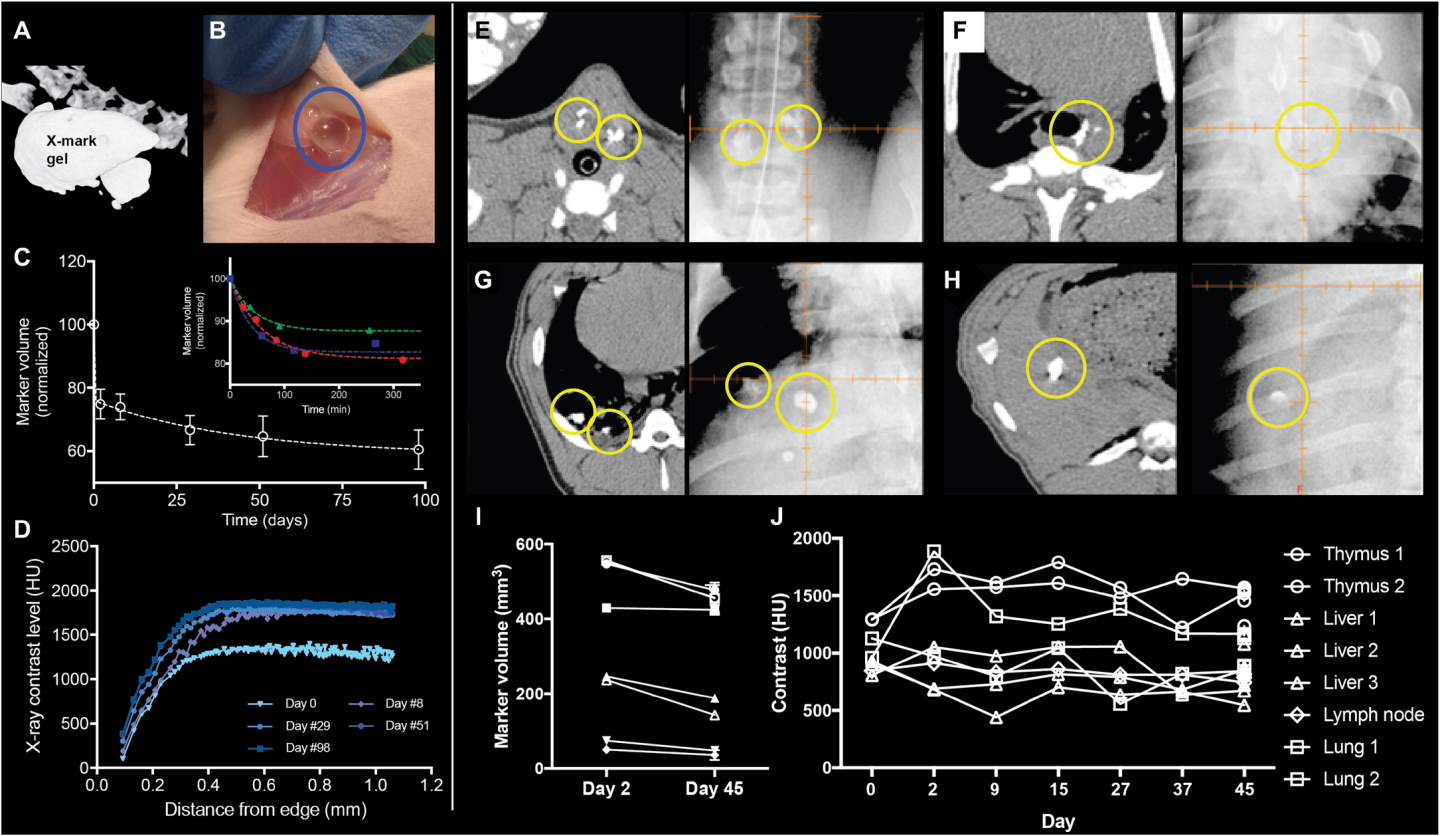
CT contrast and stability of X-mark and evaluation of performance in pigs. (**A**) Contrast and stability in mice were evaluated by injecting 50 μl of X-mark subcutaneously and CT scans performed repeatedly over a 98-day period, representative image for day 98. (**B**) Ex vivo (>99 days), the markers were transparent and well defined (Photo credit: Anders E. Hansen, Technical University of Denmark). (**C**) Volume segmentation analysis of the CT images demonstrates a gradual volume decrease over the 98-day period (*n* = 8), and an initial volume reduction caused by ethanol efflux (*n* = 3) (inset). The time required for 95% complete ethanol efflux (*T*_95%_ = 141 ± 32 min) was determined using a single exponential decay fit for each curve in the inset of (C). (**D**) The stability of radial CT contrast distribution for a representative marker is demonstrated at five time points. Feasibility and performance of X-mark in pigs. Axial CT image (left) and kV planar imaging (right) of 200 μl of X-mark (yellow circles) in the left and right lobe of the thymus injected using EBUS (**E**), 200 μl of X-mark (yellow circle) injected using EUS in an mediastinal lymph node (**F**), 200 and 500 μl of X-mark (yellow circles) injected percutaneously in the lung (**G**), and 200 μl of X-mark (yellow circles) injected percutaneously in the liver (**H**). (**I**) Volume of the segmented markers on CT performed at days 2 and 45. (**J**) CT contrast over time of segmented markers.

To investigate the imaging characteristics of X-mark, 50-μl markers were injected subcutaneously in the flanks of mice, and micro-CT imaging was performed over a period of 98 days. X-mark retained high radiodensity (>1750 HU) throughout the study (Fig. 2A), and no adverse reactions or toxicities were observed (fig. S4, A to D). Markers were easily identified as clear viscous structures ex vivo, displaying high geometrical and positional stability (Fig. 2B), which is highly important for their application as three-dimensional fiducial markers for IGRT. An initial ~20% reduction in marker volume was observed within the first hours after injection due to ethanol efflux (Fig. 2C, inset). A slow degradation of the markers was observed during the 98-day imaging period, yielding an additional marker volume reduction of approximately 15% (Fig. 2C) and an increase in radiocontrast with respect to the marker edge (Fig. 2D).

The optimal liquid marker system has equal performance across different soft tissues and injection volumes. To investigate the influence of tissue composition, ethanol efflux kinetics and coalescing properties were compared for X-mark (50 μl) injected in the subcutaneous space and adipose tissue of rats. No significant differences were identified in marker surface area (*A*_m_) 6 hours after injection (*P* = 0.33) or time required for 95% completion of ethanol efflux (*P* = 0.68) for markers injected subcutaneously (*T*_95%_ = 165 ± 45 min, *A*_m_ = 0.54 ± 0.05 cm^2^) or in adipose tissue (*T*_95%_ = 138 ± 45 min, *A*_m_ = 0.62 ± 0.05 cm^2^) when evaluated by micro-CT (Supplementary Materials; fig. S4, E to G). Marker volume may vary with different clinical situations. The influence of marker volume on efflux kinetics was, therefore, explored. Here, the mean ethanol efflux time from a 200-μl marker (*T*_95%_ = 330 ± 54 min) was significantly (*P* = 0.009) longer compared with 50 μl of X-mark (*T*_95%_ = 165 ± 45 min) (Supplementary Materials; fig. S4, E to G). This may be explained by the reduction in marker surface area–to–volume ratio, which increases diffusion length and slows ethanol efflux. However, no clinical impact on marker performance is expected from these differences.

The liquid state and imaging properties of X-mark make it attractive as an IGRT fiducial marker in, e.g., the lungs and thoracic cavity because of ease of transbronchial marker placement (Fig. 1A). Translational studies were therefore conducted in pigs, as they are the preferred animal model for translational respiratory research (*29*). Transbronchial placement and performance of X-mark markers were investigated over a 6-week study period. X-mark (200 to 500 μl) was injected by experienced thoracic surgeons using fluoroscopy- or US-guided percutaneous injections and endobronchial ultrasound (EBUS) or endoscopic ultrasound (EUS) injection. X-mark (200 to 500 μl) was injected into the thymus (Fig. 2E), mediastinal lymph nodes (Fig. 2F), lungs (percutaneous; Fig. 2G), and liver (Fig. 2H). The marker characteristics were analyzed by seven consecutive CT scans performed during the study (Fig. 2, E to H). In addition, CBCT and kilovoltage (kV) planar imaging were performed in conjunction with the second and seventh CT scans (Fig. 2, E to H). The markers formed well-defined structures with acceptable volume stability, distinct edges, high radiographic contrast, and excellent visibility across x-ray–based imaging modalities (Fig. 2, I and J, and figs. S5 and S6). These characteristics render X-mark optimal for radiography-based imaging techniques on modern IGRT equipment. The increasing use of MRI in radiotherapy treatment planning (*30*) has created the need for optimized soft tissue markers to bridge MRI coregistration to radiography-based modalities. Preceding studies in phantoms of X-mark demonstrated excellent imaging properties with low levels of image distortion on MRI in comparison to metal-based solid markers (*27*). MRI of X-mark placed in the thymus of a pig was performed postmortem. Here, the marker was identified as a signal void structure on T1- and T2-weighted images (fig. S5), potentially allowing it to serve as fiducials in both MRI- and radiography-based IGRT.

### XPV-mark: A palpable and visible surgical marker

**Fig. 3:**
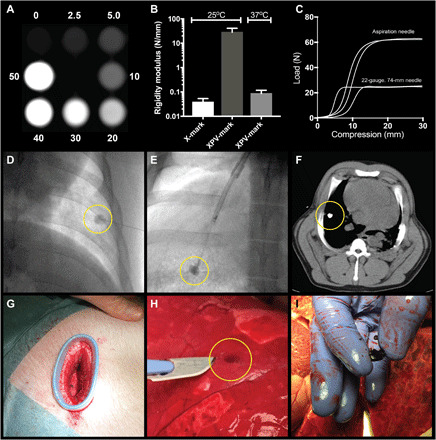
Functional performance of XPV-mark in bench testing and pigs. The CT contrast was investigated for markers containing 0 to 50% (w/w) xSAIB (**A**), and the compressibility of XPV-mark (LOIB:xSAIB:EtOH:DC2, 51.9:30:18:0.1) and X-mark (SAIB:xSAIB:EtOH, 50:30:20) was compared (**B**). (**C**) The injectability (backpressure) of XPV-mark loaded into a 1-ml syringe was determined for a 22-gauge/74-mm-length injection needle and a 21-gauge/150-cm aspiration needle. XPV-mark was injected percutaneously using fluoroscopic guidance (**D**) and by transbronchial endoscopy (**E**) into the lung of pigs. (**F**) CT imaging demonstrated that well-circumscribed and radio-dense markers were formed at the injection sites. (**G**) A thoracic port was placed to allow for inspection and palpation of the lung surface using a clinically comparable setup. The superficial markers were identified by their blue color or by palpation (**H**) and could be readily identified and separated from lung tissue (Photo credit: Jonas R. Henriksen, Technical University of Denmark) (**I**).

Localizing small nonpalpable nodular lung lesions during VATS is challenging. A surgical marker that can be positioned with high precision and aid in localizing the lesion of interest either visually or by palpation is, thus, highly warranted. On the basis of the Carbo-gel system, XPV-mark was developed as a liquid injectable soft tissue marker that renders nonpalpable lesions palpable and visible for surgical localization. The XPV-mark composition (LOIB:xSAIB:EtOH:DC2, 51.9:30:18:0.1) is based on the solidifying carbohydrate ester LOIB, the radiopaque xSAIB with addition of the hydrophobic blue color D&C Violet 2 (DC2) (Fig. 1B). This composition was investigated for injectability, palpability, CT contrast, and visibility in bench testing and in preclinical models (Fig. 3, A to I).

Upon injection, XPV-mark formed rigid markers at 25° and 37°C with a 700-fold higher resistance to compression than the SAIB-based X-mark (Fig. 3B), and XPV-mark was, therefore, chosen as the preferred palpable marker. The viscosity of the liquid marker before injection is directly dependent on both temperature and ethanol content, which affects the forces needed to shear the marker in vivo and the syringe backpressure required for injection. Therefore, the XPV-mark composition was designed to produce acceptable backpressures of <80 N to allow for injection through endoscopic aspiration needles, allowing transbronchial marker injection (Fig. 3C).

The ability of the formed rigid XPV-markers to guide localization of nonpalpable peripheral lung lesions was investigated in a proof-of-concept study in pigs. On the basis of CT and real-time fluoroscopy guidance, 300 to 600 μl of XPV-mark (LOIB:xSAIB:EtOH:DC2, 51.9:30:18:0.1) was percutaneously injected into lungs of pigs (*n* = 2) at 1 to 3 cm from the lung surface (Fig. 3D and movie S2). Injection of 300 μl of XPV-mark was also performed in the lung of pigs (*n* = 2) using a single-use bronchoscope (aScope, Ambu) and a 22-gauge bronchoscopic aspiration needle (Fig. 3E). The markers were evaluated in the caudal lung lobes, as these allowed the operator to advance the bronchoscope aspiration needle sufficiently to assure injection into lung parenchyma using the single-use bronchoscope. All injections in the caudal lung sections formed well-circumscribed markers that were clearly visible on fluoroscopy during injection. CT scans performed directly after injection and ~24 hours after injection confirmed positional stability (Fig. 3F and fig. S7) with no signs of pneumothorax or other complications. A wound retractor was placed to allow an experienced thoracic surgeon to perform digital palpation and visual inspection of an injected marker with the pig under anesthesia and artificially ventilated (Fig. 3G). To allow for palpation of percutaneous injected markers, pigs were euthanized ~24 hours after injection of XPV-mark, and immediately afterward, the lateral thoracic wall was removed to expose lung surfaces and allow for surface palpation. All markers injected into the lungs (10 of 10) could be identified by their distinct solid structure during index finger surface palpation by two experienced veterinary surgeons and two nonmedical operators. This verifies that XPV-marks’ resistance to compression is sufficient for efficient palpation in soft tissue. Furthermore, superficially located markers could be identified by the distinct dark blue color (Fig. 3H). No sign of extraction of DC2 colorant into the surrounding tissue was observed (Fig. 3I), and no complications related to the tissue markers or injection procedure were observed for any of the injections. On the basis of these observations, XPV-mark holds promise as a soft tissue marker for nonpalpable lung lesions by potentially improving intraoperative localization.

### XPVN-mark: A liquid marker for fluorescence-guided surgical procedures

**Fig. 4:**
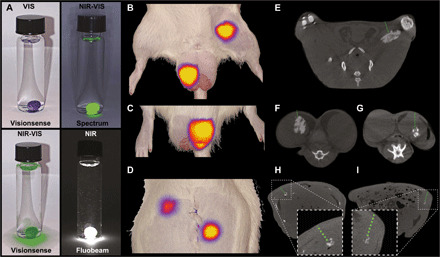
Fluorescence imaging of XPVN-mark. NIR fluorescence (NIR) and visual (VIS) light imaging of XPVN-mark (300 μl of XPVN in water) using commercially available NIR cameras: (i) Visionsense Iridium, (ii) Quest Spectrum, and (iii) Fluobeam, Fluoptics (**A**). XPVN-mark was injected into the thigh (100 μl) and right testicle (100 μl) (**B**) and left testicle (50 μl) (**C**) of a rat and NIR-VIS imaged (Visionsense Iridium). The abdominal cavity was surgically opened, and 10 μl of XPVN-mark was injected into the liver (top) and spleen (bottom) and NIR-VIS imaged (Visionsense Iridium) after surgically closing the abdominal wall (**D**). Corresponding axial-sliced CT images of the injected markers in thigh (**E**), testicles (**F** and **G**), liver (**H**), and spleen (**I**). The green dashed lines indicate the tissue depth (E: 5 mm, F: 2 mm, G: 6 mm, H: 5 mm, and I: 6 mm).

To expand the clinical application of the Carbo-gel system and further improve intraoperative localization of structures and lesions, XPVN-mark was developed by including the NIR fluorescent dye Cy7.5 covalently bound to a fully acylated sucrose derivate, Cy7.5–sucrose septaisobutyrate (SSIB) (Fig. 1B). NIR fluorescence in the 700- to 900-nm wavelength range has reduced tissue absorption, scattering, and autofluorescence (*31*). This allows for fluorescence-guided detection of soft tissue markers injected into, e.g., solitary pulmonary nodules, lymph nodes, critical tumor margins, and foreign bodies. The Cy7.5-SSIB derivate was developed as a hydrophobic analog of the U.S. Food and Drug Administration (FDA)–approved indocyanine green (*31*–*33*) and matches the spectral properties of clinical NIR cameras (*34*, *35*). The Cy7.5-SSIB derivate has excitation and emission maxima of 796 and 840 nm, respectively (Supplementary Materials; fig. S8). The optimal XPVN-mark composition (LOIB:xSAIB:EtOH:DC2:Cy7.5-SSIB, 70:10:20:0.1:0.01), with minimal self-quenching and maximal fluorescence emission intensity above 800 nm, was evaluated in experimental models. XPVN-mark provided strong NIR fluorescence emission across the tested CE and FDA-approved NIR cameras (Fig. 4A). Initial proof of concept for XPVN-mark was established by NIR imaging and CT imaging of rats. Injections were performed at varying tissue depths and volumes in the testicles, as a model for a similar-sized lymph node, and thigh musculature (Fig. 4, B to G). XPVN-mark was clearly visible at all tissue depths and volumes evaluated. XPVN-mark was also injected in the spleen and liver, and despite only injecting ~10 μl, a strong fluorescence signal was observed through the abdominal wall (Fig. 4, D, H, and I).

**Fig. 5:**
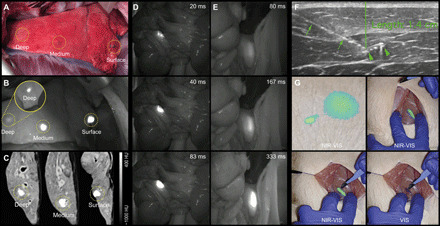
NIR fluorescence imaging of XPVN-mark in pigs. (**A**) XPVN-mark was injected (200 μl) from the medial aspect of the lung after opening the intercostal space. (**B**) Marker positions were identified by NIR imaging (Fluobeam, Fluoptics). Higher NIR intensity of the deeply positioned marker [inset in (B)] was achieved by covering more superficial markers. The distances on CT from lung surface to superficial boarders of markers were 6 and 2 mm and in the surface of the deflated lung (**C**). In addition, XPVN-mark was injected (50 μl) in a mesenteric lymph (**D**) and in the psoas muscle at tissue depth of >1 cm from the muscle surface (**E**) and imaged using various integration times (Fluobeam, Fluoptics). By US guidance, a microchip (arrowheads) was identified and the position injected (needle; arrows) with XPVN-mark (**F**). The microchip was then recovered using a commercial NIR camera with visual light (VIS) and NIR overlay (NIR-VIS) (Visionsense Iridium) (Photo credit: Jonas R. Henriksen, Technical University of Denmark) (**G**).

Proof of concept was additionally demonstrated by injection of 300 μl of XPVN-mark at varying depths in pig lungs (Fig. 5A). Here, superficially located markers could be visually identified by blue color, whereas markers positioned at deep and medium distance from the surface were nonvisible. On NIR camera imaging, all XPVN-markers were clearly visible at all three depths (Fig. 5B). On postmortem CT scans of the collapsed lung, the tissue depths of the deep and medium markers were determined to be 6 and 2 mm relative to the surface (Fig. 5C). To further explore the potential of the XPVN-marker, procedures were established to mimic challenging clinical situations in terms of accurately locating a tissue or region identified during diagnostic imaging. Identifying specific lymph nodes during surgical procedures can be highly challenging, and XPVN-mark (50 μl) was, therefore, injected into randomly selected mesenteric lymph nodes. A blinded surgeon was afterward asked to locate the injected lymph node. During the systematic evaluation of the mesentery, the injected lymph node was easily located by distinct emission of NIR fluorescence, even at short camera integration times (Fig. 5D;,fig. S9, A to F, and movie S3). The high fluorescence intensity of XPVN-mark eliminates the inherent challenges associated with low intensity and signal-to-noise of systemically administered targeted dyes and provides effective guidance even at the very limit of NIR light penetration. This was demonstrated in musculature, where a marker injected more than 1 cm from the muscle surface was clearly visible (Fig. 5E and movie S4). To mimic the clinical situation where a specific structure, e.g., a foreign body, is identified and must be surgically removed or explored, a transponder microchip was injected in the medial thigh musculature of a pig. An experienced radiologist was asked to locate the microchip by US and inject XPVN-mark to guide surgical exploration. Using a commercial NIR camera (Visionsense Iridium), a distinct signal was observed at the skin surface at the injection depth of 1.4 cm and provided accurate guidance for the surgeon during dissection and recovery of the microchip (Fig. 5, F and G, and fig. S9G). The NIR imaging properties of XPVN-mark provide a clinically relevant presurgical marking of structures, lesions, and surgical borders that can otherwise be difficult to identify. The ability to inject a positionally stable soft tissue marker with high precision and high fluorescence intensity is attractive to improve surgical localization of structures of interest, reduce surgical time, and procedure-associated trauma.

### XPVN-[^64^Cu]-mark and XPVN-[^125^I]-mark: Radionuclides provide surgical radio guidance and PET and SPECT imaging properties

**Fig. 6:**
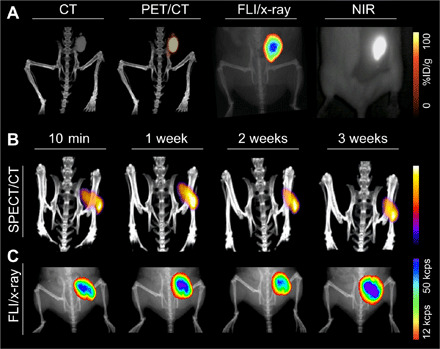
CT, PET, SPECT, and fluorescence imaging of radio-labeled XPVN-markers. In vivo evaluation of radio-labeled XPVN-markers was performed in mice. XPVN-[^64^Cu]-mark was injected subcutaneously (50 μl), and CT, PET/CT, small animal fluorescence, and surgical NIR camera (Fluoptics, Fluobeam; exposure, 13 ms) images were acquired (**A**). XPNV-[^125^I]-mark was injected subcutaneously (50 μl), and SPECT/CT multiple scans were acquired after injection (**B**) followed by FLI/x-ray imaging (**C**). ID, injected dose; cps, counts per second.

The fundamental limitations of NIR light penetration hinder long-distance guidance even for the very high fluorescence intensity provided by the XVPN-mark. To overcome this and provide optional PET or SPECT and gamma imaging/tracking possibilities, the Carbo-gel system was expanded by incorporation of radionuclides (Fig. 1, A and B). Radionuclides provide long-distance surgical guidance by use of energy-adapted handheld detectors (*36*). Long-range radio guidance and near regions-of-interest NIR guidance provide a unique tool to optimize surgical approaches and localization. This combination provides guidance all the way from incision to close-to-target fluorescence-guided dissection, which may improve accuracy and limit surgical trauma. The positron emitter ^64^Cu or single-photon emitter ^177^Lu were, to serve as examples, embedded in XPVN-mark by coordination to the ionophore, 8-hydroxyquinoline (Fig. 1B). Here, hydrophobic electroneutral complexes of either ^64^Cu(8HQ)_2_ or ^177^Lu(8HQ)_3_ were formed in ethanol and dissolved in the marker formulations. PET/CT studies in mice demonstrated high retention of ^64^Cu in XPVN-mark over a period of 42 hours (Supplementary Materials; fig. S10, A to C). The high radionuclide retention of XPVN-[^64^Cu]-mark basically eliminates signal-to-noise issues associated with systemically administered radioisotopes for surgical guidance (*23*). The high marker retention, short half-life of ^64^Cu (*T*_1/2_ = 12.7 hours), and minimal activity used (1 MBq per marker) further allow the marker to remain in the tissue until the radio emission has ceased. Radiographic contrast, stability, and volume characteristics of XPVN-[^64^Cu]-mark were identical to the preceding XPVN-marker formulations (Fig. 6A). In addition, XPVN-[^64^Cu]-mark provided high fluorescence yield on a dedicated small-animal fluorescence imaging (FLI) scanner and on a clinical NIR imaging camera (Fig. 6, A to C).

High-energy detectors for surgical radio guidance are available, but low-energy gamma emission–based radiosurgery has broader clinical applications. To include low-energy gamma emission–based radio guidance, radioiodine (^125^I; half-life, 59.5 days) was incorporated into XPNV-[^125^I]-mark via ^125^I-SSIB (Fig. 1B) using a previously described method (*37*). Briefly, a trimethylsilyl derivative of SSIB was radio-iodinated with ^125^I (Fig. 1B), producing a highly stable radiolabeled compound (^125^I-SSIB), which was dissolved in the XPVN-mark formulation. Bench testing demonstrated high retention (>95%) of ^125^I-SSIB in XPNV-[^125^I]-mark (Supplementary Materials; fig. S10D). Photon emission and iodine retention were investigated by SPECT/CT and gamma counting of markers and the thyroid of mice injected with XPNV-[^125^I]-mark. SPECT/CT imaging (Fig. 6B) demonstrated that the ^125^I activity and the radiographic contrast of the marker were maintained over a 3-week imaging period. Radio emission from the thyroid region was too low for SPECT imaging, and only 0.4 ± 0.04% of the injected ^125^I was found in the thyroid during gamma counting. In line with this, the fluorescence signal was found to be stable over the 3-week period, which demonstrates very high retention of ^125^I and NIR signal in the XPVN-marker (Fig. 6B and fig. S10, D to F). The high retention of XPNV-[^125^I]-mark infers a safe use period of at least 3 weeks between injection and surgical excision of the marker. The data (Fig. 6B and fig. S10, D to G), however, also indicate that the 3-week period may be successfully extended if required. On the basis of the imaging properties, high-precision injection technique, and the possibility to validate position across multiple imaging modalities, the XPVN-[^125^I]-mark formulation provides an attractive alternative to current ROLL markers.

## DISCUSSION

Soft tissue markers are becoming increasingly important to translate the high sensitivity of modern diagnostic imaging technologies into improved therapeutic interventions. Currently available markers are associated with important organ-dependent application and complication issues and have limitations in imaging characteristics across different modalities. Alternatives have, therefore, been clinically requested to provide accurate and safer guidance during therapeutic interventions, for example, IGRT and surgery. The Carbo-gel system was developed as a technology with excellent imaging characteristics and minimal invasiveness due to its liquid state before injection. Injection is possible even through long endoscopic aspiration needles, and X-mark (Carbo-gel) markers displayed good radiocontrast and maintained positional stability throughout a standard period of fractionated radiotherapy (months). The advantage of the marker technology has been demonstrated in patients with lung cancer (*25*). The liquid Carbo-gel technology may open options for marking of anatomical locations where current technologies may be difficult to apply. New indications for Carbo-gel are currently being explored. Marking of esophageal neoplasms has been demonstrated (*38*), and clinical investigations are currently under way in patients with bladder and rectal cancer (clinicaltrials.org-NCT03265418). In addition, lower marker-related dose perturbations for proton therapy have been demonstrated for X-mark in comparison to current metallic markers, which currently limits the use of intratumoral markers for heavy beam therapy (*5*, *39*). An inherent limitation for tissue markers in general is the ability of the surgeons to accurately inject the marker. The thin-needle injectability and imaging properties of Carbo-gel allow surgeons to perform, e.g., fluoroscopy-, CT-, or ultrasound-guided injections using high-precision clinical technologies. After injection, the position of the marker can be confirmed by CT, MRI, and even PET or SPECT imaging. This secures that an optimal therapeutic procedure with enhanced precision can be performed with real-time guidance using Carbo-gels’ palpatory properties or by NIR or radio emission.

Through the development of the Carbo-gel system, a number of clinical applications have emerged beyond image-guided radiotherapy, which underlines the need for marker technologies that can bridge diagnostic and therapeutic procedures. The design of the solidifying blue-colored XPV-mark enabled formation of palpable structures in the lungs of pigs that may assist in accurately identifying, otherwise occult and impalpable, lung regions or lesions of interest during VATS. With XPV-mark, smaller lesions and early-stage lung cancers may be identified and resected earlier with obvious benefit to the patient.

Fluorescence-guided surgery is the subject of intense research, and attractive technologies for marking cancer cells with systemically administrated fluorophores are being investigated (*1*, *35*). Despite the ability to directly label cancerous tissues, this strategy may have inherent limitations. The NIR fluorescent light penetration from labeled cells is potentially shorter than the optimal required margins in cancer surgery. Furthermore, the low fluorophore concentration and, hence, fluorescence intensity for systemically or locally administered dyes can only be visualized at low tissue depths using current NIR-I fluorophores. Carbo-gel provides a versatile technology wherein the fluorescent dye is retained at the site of injection, providing higher fluorescence intensity and thereby enables marker identification deeper into tissues. Physicians may place multiple markers in, or around, lesions of interest or regions where care needs to be taken to avoid fragile structures, minimize trauma, or secure excision margins. The multimodal imaging possibilities and the use of nontargeted labels, in general, allow for high-precision image-guided injections and thereby provide surgical guidance independent of patient type, disease, or receptor or ligand expression levels. In the case of Carbo-gel, the surgeon may use fluorescence from XPVN-mark placed at the desired surgical borders as positive guidance to secure clean margins. This is contrary to cancer cells labeled by targeted fluorophores, where NIR light (based on tissue penetration <1 cm) can be a negative indicator of being too close to cancerous tissue margins during resection. Although very high amounts of fluorophore and even next-generation NIR-II dyes can be incorporated in Carbo-gel, which for XPVN-mark enabled guidance at >1.4-cm tissue depths, fluorescent light is attenuated by tissue, which limits guidance distances. The flexibility to incorporate radionuclides in Carbo-gel eliminates this problem, as gamma emissions travel sufficient distances for all locations in patients. In addition, the radiolabeled markers demonstrated excellent retention of radionuclides in vivo, as well as conservation of NIR fluorescence and NIR imaging properties. This attests to a lack of substantial in vivo instability as a result of radiolysis. XPVN-[^64^Cu]-mark or XPVN-[^125^I]-mark, therefore, allows for optimal surgical approaches in conventional, image-guided, and robotic surgery. Specifically, robotic surgery may benefit from image-guided planning based on presurgical multimodal radioactive markers. The injected radioactive markers may here serve as accurate internal intraoperative navigation points, providing a GPS-like guidance based on presurgical image maps that can secure accurate surgical navigation with optional NIR guidance at short range. The versatility of the marker system makes it highly advantageous toward the addition of radioisotopes, thereby securing optimal imaging properties. Furthermore, all radiolabeling of a preformulated Carbo-gel can be performed at an in-hospital radiopharmacy using standard equipment. This allows all radionuclide handling to be performed according to institutional and national radioisotope guidelines, which is highly attractive from a production, distribution, storage, and regulatory point of view. The radioactivity levels included in this study have already been reported as safe for patients and medical personnel in breast surgery (*40*). The use of radio guidance must, however, be evaluated in terms of acceptable radiation dose for each indication and adhere to applicable guidelines.

The Carbo-gel system uses an innovative technology that allows for step-by-step addition of imaging properties. The highly encouraging results demonstrate that the technology may provide simple solutions to challenging clinical problems by bridging advanced high-resolution diagnostic imaging to therapeutic intervention.

## MATERIALS AND METHODS

### Study design and animal handling

The aim of this study was to develop and evaluate the Carbo-gel multimodality fiducial marker technology across translational models. Sample sizes for statistical analysis on mice and rat study parts were determined by power calculations based on previous work. The subsequent evaluation of marker performance sample sizes was based on logistical and ethical consideration to achieve validity of obtained results. The 24-hour CT scan of two rats in the evaluation of tissue influence on ethanol efflux had to be excluded because of technical problems during CT acquisition. The study was designed as controlled laboratory experiments with the objective of evaluating the liquid multimodal marker system performance and clinical potential across a number of translational models. For studies involving comparisons, animals were randomly assigned to groups.

All procedures were approved by the National Animal Research Inspectorate and the Institutional Ethical Board. Mice and rats were anesthetized using sevoflurane (3 to 5%) or isoflurane (2 to 3%) in oxygen/air gas mixture, and pigs by intramuscular injection of tiletamine/zolezepam and maintained using a continuous infusion of propofol or sevoflurane (1 to 3%) in oxygen/air gas mixture. Pigs had endotracheal tubes placed, and a mechanical respirator was used as needed. Pigs were euthanized by intravenous injection of pentobarbital.

### Preparation of multimodal markers and synthesis of Carbo-gel materials

Marker formulations are homogeneous liquids and are prepared by mixing of components in closed vials followed by sonication, magnetic stirring, and heating. Detailed description of the preparation of X-mark, XPV-mark, XPVN-mark, and radioactive analogs and the synthesis of LOIB, xSAIB, and Cy7.5-SSIB are described in detail in the Supplementary Materials.

### In vivo stability and tolerability of X-mark in mice

Seven-week-old female Naval Medical Research Institute (NMRI) mice (Taconic, Lille Skensved, Denmark) were clipped and aseptically prepared over the right flank for injection of X-mark [SAIB:xSAIB:EtOH (50:30:20), *n* = 8] or control markers [SAIB/EtOH (80:20), *n* = 8]. X-mark (50 μl) was injected subcutaneously using a 1-ml syringe and 25-gauge/25-mm needle. Blood samples were collected before injection of X-mark, or control marker, and at multiple time points. Serum cytokine markers tumor necrosis factor–α, interferon-γ, and interleukin-6 were analyzed using a bead-based sandwich immunoassay and a mouse cytokine panel kit (catalog no. MCYTOMAG-70K-03/Luminex LX100, Millipore, Burlington, MA, USA) (Supplementary Materials and Methods and fig. S4, A to D).

Hounsfield-corrected CT scans (67 kVp, 500 μA, 360 rotation steps, exposure of 400 ms, voxel size of 0.092 × 0.092 × 0.092 mm) were recorded directly after injection of the marker and repeatedly over a period of 14 weeks at days 0, 2, 8, 29, 51, and 98 (Inveon PET/CT, Siemens, Malvern, PA, USA). Three additional mice had multiple CT scans performed during the first 3 hours after injection to investigate initial X-mark EtOH efflux kinetics.

X-mark was semiautomatically segmented using a Chan-Vese model (*41*) using an in-house written script in MATLAB 2013a (MathWorks, Natick, MA, USA). A bounding box was drawn around the marker in all dimensions and was automatically segmented into marker/nonmarker. The initial estimate of the marker for automatic segmentation was based on the 99th percentile of the maximum contrast value (HU) in the bounding box. Bony structures were excluded if present inside the bounding box. The segmented markers were analyzed in terms of marker volume and time.

### Imaging performance of X-mark in pigs

Studies were performed using female pigs (~45 kg, *n* = 4). X-mark was injected using CT, US-guided, or unguided percutaneous injection (63/90-mm, 22-gauge needle) or EBUS (Olympus BF-UC180F EBUS-scope and EZ Shot 2 EUS 22-gauge needle) and using EUS (Olympus GF-UCT140-AL5 EUS-scope and EZ Shot 2 EUS 22-gauge needle). Markers were monitored real time during injections or after by C-arm fluoroscopy (OEC-09TH, GE Healthcare, Chicago, IL, USA) using automatic exposure control (74 to 95 kVp, 2.64 to 3.0 mA). X-mark was injected in the thymus (200 μl, EBUS), mediastinum (200 μl, EUS), liver (200 to 300 μl, percutaneous ultrasound and EUS), lung (percutaneous, 200 and 500 μl), and mediastinal lymph nodes (200 μl, EBUS). CT scans were performed immediately after injection [Somatom Emotion Siemens, Erlangen, Germany; 110 kVp/140 to 200 mAs/2 mm, 400 mm per field of view (FOV), H31s]. Two pigs were euthanized after 24 hours to evaluate injected regions. A postmortem MRI scan (Biograph mMR PET/MR 3T Siemens, Erlangen, Germany) using T1/T2 in-house–developed sequences was performed of the thymus region in one pig.

Six-week stability and visibility were evaluated in two pigs. CT scans were performed at days 0, 9, 15, 27, and 37 [Somatom Emotion Siemens (identical settings as above)] and on days 2 and 45 (Somatom Definition AS Siemens, Erlangen, Germany; 120 kVp/100 mAs, 1 mm per slice, 400 mm per FOV). Fluoroscopy (OEC-09TH) was performed on days 0, 9, 27, and 37. CBCT (TrueBeam STx Varian, Palo Alto, CA, USA; 125 and 100 kVp) and low-dose on-board imaging (OBI) fluoroscopy (66 to 77 kVp, 40 to 57 mAs) were performed on days 2 and 45. For the CT evaluation, mean contrast and volume were investigated. All measurements of HU and contouring were performed in MATLAB 2013a or Osirix 5.7. A maximum-intensity projection was used for three directions to select the marker for evaluation. Three-dimensional regions of interest (ROIs) containing the marker was constructed, and a lower contouring threshold of 400 HU was selected for delineation and corrected for obvious structures, e.g., bone in the contouring volume. Fluoroscopy was evaluated by visual inspection of the images to determine marker visibility.

### Bench testing of XPV-mark

#### X-ray visibility of XPV-mark

Liquid marker formulations with 0, 2.5, 5.0, 10, 20, 30,40, and 50% (w/w) xSAIB and fixed amounts of EtOH (18.0%, w/w) and DC2 (0.10%, w/w) were prepared using the general procedure. Liquid marker formulations (200 μl) were added to separate wells in a 96-well microtiter plate. Evaluation of radiopacity of the liquid marker formulations as a function of xSAIB concentration was performed using CT (120 kVp, 300 mAs, 200-mm FOV, and extended CT scale, B30s, Siemens Somatom Definition AS). Contrast was quantified using Eclipse 13.7 (Varian, Palo Alto, CA, USA).

#### Marker compressibility

Markers were prepared with a consistent size and geometry by injection of 300 μl of liquid marker formulation into glass vials containing MQ-H_2_O (20 ml, 50°C). Subsequently, the vials were incubated at 37°C, after which the water was replaced at 24 and 72 hours, respectively. Compression was investigated using an Instron mechanical tester (Instron 5967) equipped with a 50-N load cell. The markers were submerged in temperature-controlled (25° or 37°C) MQ-H_2_O inside a transparent thermostat jacket cell during compression. For each experiment, eight markers were mechanically tested by monitoring the load (N) as function of the compression (mm). The marker modulus was determined by the slope of the load-versus-compression curve for its initial linear part.

#### Syringe backpressure

The syringe backpressure was determined for the XPV-mark formulation using an Instron mechanical tester (Instron 5967) equipped with a 500-N load cell and a syringe module (OP104479-1001). The backpressure was determined for a 1-ml luer-lock syringe equipped with a 22-gauge hypodermic needle (*l* = 74 mm) or 21-gauge electromagnetic navigational bronchoscopy (ENB) aspiration needle (superDimension *l* = 144 cm, Medtronic, Dublin, Ireland,). The syringe content was ejected at 1 mm/s, and the corresponding backpressure/force and compression was recorded.

### Evaluation of XPV-mark as surgical marker in pigs

Female pigs (~45 kg, *n* = 4) had a CT scan (130 kVp/140 to 200 mAs/2 to 3 mm, H31s medium smooth, Somatom Emotion) performed on day 0 to guide injections of XVP-mark. XVP-mark was injected using CT-guided percutaneous (*n* = 2) injection (22-gauge/63-mm needle) or using a single-use bronchoscope (*n* = 2). For percutaneous injections, the injection volumes were 600 or 300 μl bilaterally in the lungs. Two to three markers were injected in each lung to secure sufficient intermarker distance during lung palpation. Markers were visualized real time during injections by C-arm fluoroscopy (OEC-09TH, 74 to 95 kVp, 2.64 to 3.0 mA) (Supplementary Materials; fig. S7). Directly following injections, pigs were CT scanned and again 24 hours after injection. Pigs were subsequently placed in lateral recumbency and euthanized. The thoracic wall was surgically removed to allow for palpation of the lung surface on one side followed by the opposite side. Lungs were visually inspected, and palpation strictly by index finger was performed by four independent operators (two veterinary surgeons and two inexperienced nonmedical persons). All operators scored injected markers as visually identifiable on the surface of the lung (yes/no) and by palpation (yes/no/maybe). Palpation “maybe” score was defined as an assessment result where the operator can feel a structure but would not pursue surgical removal without additional diagnostics. For all palpatory procedures, lung temperature was measured by a temperature probe placed at 1-cm depth in the lung tissue. Lungs were subsequently removed from the thoracic cavity and CT scanned to investigate whether the locations identified were actually the injected markers. The possibility to perform XPV-mark injections using a bronchoscopy procedure was investigated in two pigs. Injection of XPV-mark was performed using a single-use video bronchoscope (aScope, Ambu, Ballerup, Denmark) and a bronchoscope aspiration needle. Injections were performed in the caudal lung sections. Pigs were CT scanned as described above. After completion of the 24-hour CT scan, a thoracic port was inserted in one pig, and the lung surface was palpated in one of the injected regions.

### Bench and in vivo testing of XPVN-mark

#### Bench testing

Solid markers were formed by injection of 300 μl of XPVN-mark (LOIB:xSAIB:EtOH:DC2:Cy7.5-SSIB, 70:10:20:0.1:0.01) into a glass vial containing water. The marker was incubated >24 hours at room temperature. The blue solidified XPVN-marker was then imaged on three NIR cameras: VisionSense Iridium (Philadelphia, PA, USA) (laser excitation, 785 nm; emission, 825 to 850 nm), Quest Spectrum (Middenmeer, The Netherlands) (light-emitting diode excitation; emission, 830 to 1000 nm; 38-ms integration time), and Fluobeam 800, Fluoptics (Grenoble, France) (laser excitation, 750 nm; emission, >800 nm; long-pass filter, 43 ms).

#### NIR and CT imaging performance of XPVN-mark in rats

Male Wistar rats (~400 g, *n* = 2) were euthanized and injected (23-gauge/25-mm needle) with XPVN-mark (LOIB:xSAIB:EtOH:DC2:Cy7.5-SSIB, 70:10:20:0.1:0.01). The testes were used as a model of a lymph node, and 100 and 50 μl of XPVN-mark were injected from the opposite side with respect to FLI. In addition, 100 μl of XPVN-mark was injected in the thigh musculature, and ~10 μl was injected in the spleen and liver. FLI was performed (Visionsense Iridium), and position and radiographic contrast were evaluated by CT (NanoScan SPECT/CT, Mediso, Budapest, Hungary; 50 kVp, 520 μA, 480/steps, 300 ms per exposure time).

### XPVN-mark as surgical marker in pigs

Female pigs (~55 kg, *n* = 4) were euthanized after a surgical upper airway procedure for investigation of XVPN-mark performance as a surgical marker in two clinically relevant situations. XVPN-mark was injected (200 μl, 23-gauge/25-mm needle) at three locations from the medial side of the right lung of one pig after surgically opening the chest to allow for inspection of the lung surface. Markers in lungs were evaluated by visual inspection, NIR imaging (Fluobeam), and palpation and CT scanned to accurately determine location of markers. In the subsequent pigs, the possibility to identify the marker under various conditions was evaluated. Fluorescence imaging was performed using commercially available NIR cameras (Fluobeam and Iridium). Light penetration at tissue depth of >1 cm was investigated by injecting the psoas musculature from the dorsal aspect with 100 μl of XVPN-mark (23-gauge/40-mm needle). The XPVN-mark emission was investigated from the abdominal side by performing a laparotomy with the pig placed in dorsal recumbence and dissection of XPVN-mark performed. XVPN-mark (50 μl) was also injected in mesenteric lymph nodes (23-gauge/40-mm needle), and after repositioning of the intestines, an experienced veterinary surgeon was asked to identify the injected node using NIR camera guidance. To further evaluate performance of NIR-guided dissection in the musculature, a microchip was injected in the thigh musculature of a pig, and an experienced US radiologist was asked to locate the microchip and inject (50 μl, 21-gauge/76-mm needle) XPVN-mark directly adjacent to the microchip. A blinded surgeon subsequently recovered the microchip using NIR guidance.

### In vivo evaluation of XPVN-[^64^Cu]-mark and XPVN-[^125^I]-mark

The right flank of 20-week-old female NMRI mice or 12-week-old female BALB/c mice were clipped and aseptically prepared for subcutaneous injection (50 μl, 23-gauge/40-mm needle) of XPVN-[^64^Cu]-mark (A[^64^Cu] 20 MBq/ml) or XPVN-[^125^I]-mark (A[^125^I] 41.4 MBq/ml). After the injection, a PET/CT (Inveon) or SPECT/CT scan (NanoScan) was performed. PET scans were performed 10 min, 1 hour (5-min acquisition), 17 hours (10-min acquisition), and, last, 42 hours (15-min acquisition) after injection of XPVN-[^64^Cu]-mark. PET emission data were corrected for dead time, decay, and attenuation and reconstructed using maximum a posteriori reconstruction algorithm. SPECT scans (TT3D dynamic range pinSPECT [^125^I]; medium scan, 20 frames/90 s) were performed over the injected region: 10 min, 7, 14, and 21 days after injection of XPVN-[^125^I]-mark. Image evaluation was performed using commercially available software [Inveon, Siemens, Malvern, PA, USA (PET/CT) and ViVoQuant, InviCRO, Boston, MA, USA (SPECT/CT)].

FLI was performed using a surgical NIR camera (Fluobeam; XPVN-[^64^Cu]-mark) and a small animal FLI/x-ray system (IVIS Lumina XR, Caliper Life Sciences, Hopkinton, MA, USA; XPVN-[^64^Cu]-mark and XPVN-[^125^I]-mark). FLI was performed 1, 17, and 42 hours after injection of the XPVN-[^64^Cu]-mark. FLI/x-ray imaging (binning; 2, 10-cm FOV, automatic exposure time, excitation: 745 nm, and emission: 810 to 875 nm) was performed 18 and 44 hours after injection of XPVN-[^64^Cu]-mark and directly after the XPVN-[^125^I]-mark SPECT/CT scans. Fluorescence was evaluated by manually constructing an ROI covering the gel area and recording ROI flux.

### Statistical and image analyses

Statistical analyses were performed in GraphPad Prism 7 and SAS 9.4. A *P* value of <0.05 was considered statistically significant. All data are reported as mean ± SEM, unless otherwise stated. Normal distribution of data for statistical analysis was tested by Shapiro-Wilk test and inspection of Q-Q plots. Image analyses and contouring were performed using Osirix 5.7 or MATLAB 2013a, unless otherwise stated.

## Supplementary Material

abb5353_Movie_S4.mp4

abb5353_Movie_S2.avi

abb5353_SM.pdf

abb5353_Movie_S3.mov

abb5353_Movie_S1.mov

## SUPPLEMENTARY MATERIALS

Supplementary material for this article is available at http://advances.sciencemag.org/cgi/content/full/6/34/eabb5353/DC1

## Appendix

View/request a protocol for this paper from *Bio-protocol*.
