# Supplementary material for: Multimodal soft tissue markers for bridging high-resolution diagnostic imaging with therapeutic intervention
Source: Sci Adv. 2020 Aug 19;6(34):eabb5353. doi: 10.1126/sciadv.abb5353 (PMC7438096; doi:10.1126/sciadv.abb5353)
Supplement: abb5353_SM.pdf [file abb5353_SM.pdf]

Science Advances
AAAS

advances.sciencemag.org/cgi/content/full/6/34/eabb5353/DC1

Supplementary Materials for

Multimodal soft tissue markers for bridging high-resolution diagnostic imaging with therapeutic intervention

Anders E. Hansen, Jonas R. Henriksen, Rasmus I. Jølck, Frederikke P. Fliedner, Linda M. Bruun, Jonas Scherman, Andreas I. Jensen, Per. Munck af Rosenschöld, Lilah Moorman, Sorel Kurbegovic, Steen R. de Blanck, Klaus R. Larsen, Paul F. Clementsen, Anders N. Christensen, Mads H. Clausen, Wenbo Wang, Paul Kempen, Merete Christensen, Niels-Erik Viby, Gitte Persson, Rasmus Larsen, Knut Conradsen, Fintan J. McEvoy, Andreas Kjaer, Thomas Eriksen, Thomas L. Andresen*

*Corresponding author. Email: tlan@dtu.dk

Published 19 August 2020, *Sci. Adv.* **6**, eabb5353 (2020)
DOI: 10.1126/sciadv.abb5353

The PDF file includes:

Supplementary Materials and Methods
Preparation of markers detailed material and methods
Figs. S1 to S10
Legends for movies S1 to S4

Other Supplementary Material for this manuscript includes the following:

(available at advances.sciencemag.org/cgi/content/full/6/34/eabb5353/DC1)

Movies S1 to S4

Supplementary Materials:

Preparation of markers detailed material and methods

Preparation of X-mark: Radiopaque formulations containing SAIB, xSAIB and EtOH was prepared in differing ratios. As an example, SAIB was heated to 70 ºC, and 5 g SAIB was poured into a glass vial. 3 g of xSAIB and 2 g of EtOH was mixed with SAIB and sonicated for 30 minutes to obtain a transparent and homogeneous SAIB formulation with the final composition SAIB:xSAIB:EtOH 50:30:20 %w/w. Formulations containing varying amounts of EtOH or xSAIB were prepared using similar procedure.

Preparation of XPV-mark: Radiopaque formulations forming solid and visible markers at the site of injection containing LOIB, xSAIB, DC2 and EtOH was prepared in differing ratios. The neat compounds were weighed into a glass vial, and the mixture was sonicated and heated at 70 ºC until the solution was homogeneous. As an example, LOIB:xSAIB:EtOH:DC2 51.9:30:18:0.1 was prepared for animal studies.

Preparation of XPVN-mark: NIR fluorescent formulations based on the novel fluorophore Cy7.5-SSIB were prepared by direct solubilization in XPV-mark (LOIB:xSAIB:EtOH:DC2 70:10:20:0.1). A 2 mg/mL stock solution of CY7.5-SSIB was prepared in EtOH. Following, 0.1 mL Cy7.5-SSIB stock solution was pipetted into a glass vial, and dried at 55 ºC under a gentle stream of N_2. 2 g of the XPV-mark formulation was added, and the solution was sonicated for 6 h, to obtain a final dye concentration of 0.01% w/w and final composition LOIB:xSAIB:EtOH:DC2:Cy7.5-SSIB 70:10:20:0.1:0.01. Formulations containing 0.01%, 0.006%, 0.003%, 0.001%,0.0006%, 0.0003%, 0.0001% or 0.00001% Cy7.5-SSIB were prepared by serial dilution.

Preparation of XPVN-[^{125}I]-mark: XPVN-mark was radiolabeled with ^{125}I for SPECT imaging and gamma detection by direct solubilization of ^{125}I-SSIB in XPVN-mark (LOIB:xSAIB:EtOH:DC2:Cy7.5-SSIB 70:10:20:0.1:0.01). In this procedure, the marker was radiolabeled by ^{125}I using the SSIB-TMS substrate synthesized according to the previously described procedure (Schaarup-Jensen et. al. Injectable iodine-125 labeled tissue marker for radioactive localization of non-palpable breast lesions, Acta Biomaterialia 65 (2018), p. 197-202), thereby creating a marker that is visible in SPECT/CT/NIR, that have colour and is visible by the eye, and that forms a solid which is palpable in surgical procedures.

TLCs were run in heptane:EtOAc (6:4) and developed with a $KMnO_4$ stain. SSIB-TMS eluted at Rf = 0.6 and [^{125}I]SSIB-I slightly below. Radio-TLCs were analyzed on a Cyclone Plus Storage Phosphor System (Perkin Elmer). Radioactivities were measured on a Veenstra Instruments dose calibrator VDC-505 in standardized 4 mL glass vials that had been pre-calibrated using the I-125 specifications given by Perkin-Elmer.

Radioiodination: $Tl(CF_3COO)_3$ (10.2 mg) was dissolved in a mixture of acetonitrile (2.30 mL) and trifluoroacetic acid (1.50 mL). An aliquot of this solution (380 µL) was transferred to an HPLC vial ($Tl(CF_3COO)_3$: 1.1 mg, 1.8 µmol). To the vial was then added SSIB-TMS in acetonitrile (120 µL, 1.2 µmol). The solution was stirred at RT for 2 hours. To the mixture was then added [^{125}I]NaI in 10^{-5}M aq. NaOH (30 µL, 96.5 MBq). After 43 minutes of stirring at RT, aq. NaI (18 µL, 3.6 µmol) was added, followed by 60 minutes of stirring at RT.

To the reaction mixture was added water (500 µL). This was followed by capture of the product on a SEP-PEAK C18 Plus cartridge that had been pre-washed with ethanol (2 x 5 mL) and water (3 x 5 mL). The reaction vessel and cartridge were washed with aq. DTPA (3 x 1 mL, 50 mM, pH 7.0), water (3 x 10 mL) and 25% (v/v) ethanol in water (2 x 2 mL). The product was subsequently eluted in ethanol (3 x 1 mL). The two initial fractions (2 x 1 mL) of ethanol contained the bulk of the

product. Radiochemical purity was determined in these two fractions by radio-TLC to be 90.6% (fraction 10, 51.6 MBq) and 92.6% (fraction 11, 30.0 MBq). The product was analyzed by TLC and found to be chemically pure with the identity of the radiolabeled product confirmed by comparing with the Rf of non-radioactive SSIB-I. The two fractions were pooled with 500 µL extra ethanol added to rinse to containers. The radioactive yield was determined to be 83.4 MBq (RCY: 86%).

Formulation of XPVN-[^{125}I]-mark: From the combined product fractions in ethanol (2.5 mL) was removed 2.2 mL (73 MBq), which was evaporated to dryness in a glass vial for 74 minutes at 40 ºC under at stream of argon. To the dry residue was added 1.8 mL LOIB:xSAIB:EtOH:DC2:Cy7.5-SSIB marker solution, followed by magnetic stirring at RT for 30 minutes. This gave the finished marker formulation.

Preparation of XPVN-[^{64}Cu]-mark: In this preparation, the marker LOIB:xSAIB:EtOH:DC2:Cy7.5-SSIB (70:10:20:0.01:0.01) was radiolabelled by ^{64}Cu using the hydrophobic chelator 8-hydroxy-quinoline (8HQ), thereby creating a marker that is visible in PET/CT/NIR, that have colour and is visible by the eye, and that forms a solid which is palpable in surgical procedures.

^{64}Cu production: ^{64}Cu was produced on a PETtrace cyclotron (GE Healthcare, Milwaukee, WI, USA) equipped with a beamline by proton irradiation of an electroplated ^{64}Ni target, then purified by anion exchange chromatography in aqueous hydrogen chloride (HCl) media. The ^{64}Cu was ultimately obtained in aqueous HCl (1.0 M), and isolated by evaporation of aqueous HCl by argon flow. The dry $^{64}CuCl_2$ was used for radiolabelling markers.

Radiolabelling of the marker (Fig. S1, scheme 1): A solution of 8HQ in ethanol (200 µL, 500 µM) and pure ethanol (300 µL) was mixed with dry [^{64}Cu]$CuCl_2$ (300 MBq) and stirred at room temperature, 400 rpm, for 18 hours. Ethanol solvent was evaporated at 50°C using argon flow for 20 minutes. Then 1 mL of marker formulation (LOIB:xSAIB:EtOH:DC2:Cy7.5-SSIB 70:10:20:0.1:0.01) was added to the dry film of ^{64}Cu(8HQ) and hereafter the formulation was stirred at 50 °C, 400 rpm for 2 hours. The radiolabelled marker formulation (660 µL) was transferred to a new glass vial and the radioactivity was measured by dose calibrator (Comecer, VDC-505). Non-radioactive gel formulation (1.5 mL) was added to dilute the formulation to 20 MBq/mL. The final formulation was homogenized by further stirring at 50 °C, 400 rpm for 20 minutes and vortexing.

Scheme 1:

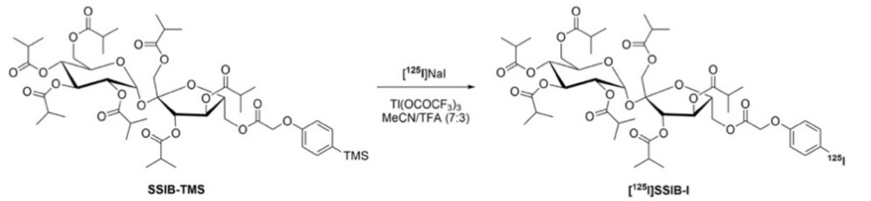

Scheme 2:

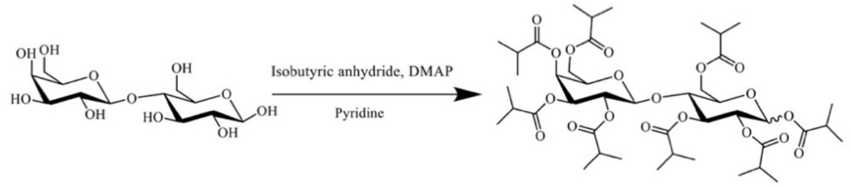

Scheme 3:

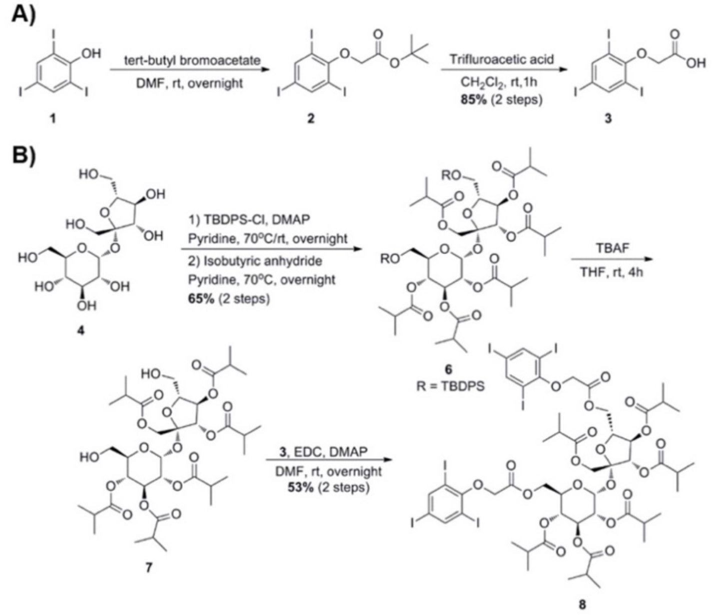

Scheme 4:

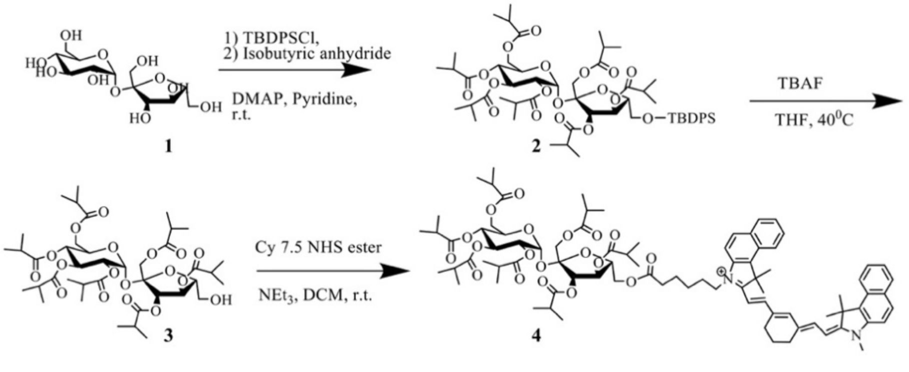

Fig. S1. Chemical reaction schemes. Scheme 1: Radio iodination of SSIB-TMS, scheme 2: Synthesis of Lactose octaisobutyrate (LOIB), scheme 3: Synthesis of xSAIB. A) Synthesis of TIPA (**3**) over two synthetic step starting from 2,4,6-triiodophenol (**1**); B) Synthesis of xSAIB (**8**) over five synthetic steps starting from sucrose (**4**), scheme 4: Reaction scheme for the synthesis of Cy7.5-SSIB.

Synthesis of the LOIB, xSAIB and Cy7.5-SSIB platform materials

α,β-Lactose octaisobutyrate (LOIB) (Fig. S1, scheme 2): β-Lactose (50 g, 146 mmol) was suspended under inert atmosphere in 500 mL of dry pyridine. Then, isobutyric anhydride (426 mL, 2.6 mol, 2.2 eq. pr. OH) was added followed by addition of a catalytic amount of DMAP (1.2 g, 9.8 mmol, 0.1 eq). The reaction was stirred for 1.5 day at 48 ºC under N_2 atmosphere, whereafter TLC (10% acetone, toluene, Rf products ~0.6) showed the reaction was completed. The reaction mixture was concentrated *in vacuo* followed by co-evaporation with toluene (2× 100 mL) to remove pyridine and most of the excess anhydride. Then followed dissolution of crude product in 1 L of chloroform and washing of the organic phase with first $NaHCO_3$ (aq) (5× 1 L) and then water (2× 1L). The organic phase was dried with $MgSO_4$ (s), filtered, concentrated under reduced pressure and dried *in vacuo*. Yield: 118.5 g (89.8 %) (~30% alpha, ~70 % beta, seen from H^1-alpha/beta NMR integral ratio, the integral of H^1-beta is by default set to 1). MALDI-TOF MS (DHB+Na): Calc $[M+ Na]^+$: 926.02. Found: 925.70. TGA (30-300^0 C, 10^0 C ramp/minute): material is completely dry. ^{1}H-NMR (400 MHz, Chloroform-*d*) δ 6.26 (d, *J* = 3.8 Hz, 0.4 H, H-1 α), 5.68 (d, *J* = 8.3 Hz, 1H, H-1 β), 5.48 (dd, 10.3, 9.2 Hz, 0.4 H), 5.40 – 5.33 (m, 2H), 5.27 (t, *J* = 9.5 Hz, 1H), 5.18-5.00 (m, 3H), 5.01 – 4.92 (m, 2H), 4.50 – 4.39 (m, 3H), 4.23-4.03 (m, ~4H), 3.95 (ddd, *J* = 10.1, 3.8, 1.7 Hz, 0.4H), 3.92 – 3.80 (m, 3H), 3.70 (ddd, *J* = 9.9, 4.3, 1.9 Hz, 1H), 2.70 – 2.32 (m, ~11H, CH isobutyrate), 1.24 – 1.01 (m, ~68H, CH_3 isobutyrate) .

Synthesis of xSAIB

2-(2,4,6-Triiodophenoxy)acetic acid (**3**) (Fig. S1, scheme 3). 2,4,6-Triiodophenol (**1**) (10.00g, 21.2mmol) was dissolved in dry DMF (75mL) under N_2-atmosphere. To this solution, *tert*-butyl bromoacetate (4.20 mL, 28.46 mmol) and K_2CO_3 (8.79 g, 63.6 mmol) were added and the stirred overnight at rt. The solvent was removed *in vacuo* and the remaining yellow oil re-dissolved in EtOAc (150 mL) and washed with MQ-H_2O (3×150 mL). The organic phase was dried with $MgSO_4$, filtrated and concentrated *in vacou* to give *tert*-butyl 2-(2,4,6-triiodophenoxy) acetate (**2**) as a light yellow oil which was used in the next step without further purification. **2** was dissolved in CH_2Cl_2 (60 mL) and trifluoroacetic acid (30 mL) was added. The mixture stirred for 1h at room temperature after which the solvent was removed *in vacuo* to give a white solid. The crude product was re-crystallized from EtOH to give 2-(2,4,6-triiodophenoxy)acetic acid (**3**) as fine white needles (9.58 g, 85% (2 steps)). ^{1}H-NMR (300MHz, MeOD): δ 6.58 (s, 2H), 2.95 (s, 2H). MALDI-TOF MS (DHB+Na): Chemical Formula: $C_8H_5I_3NaO_3$, calculated mass; 552.83; found: 553.08 ($M+Na^+$).

6,6'-TBDPS-sucrose (**5**), **6,6'-TBDPS-isobutyric-sucrose** (**6**) and **6,6'-OH-isobutyric-sucrose** (**7**)

6,6'-TBDPS-sucrose (**5**) (Fig. S1, scheme 3). Sucrose (**4**) (3.00g, 8.76mmol) was dissolved in dry pyridine (54.0mL) under N_2-atmosphere. To this solution *tert*-butyldiphenylchlorosilane (TBDPS-Cl) (2.51mL, 9.64mmol) and a catalytic amount of DMAP (107.5mg, 0.88mmol) were added and the mixture heated at 70°C for 3h. After cooling to rt, TBDPS-Cl (2.51mL, 9.64mmol) was added and the mixture stirred overnight at rt. The solvent was removed *in vacuo* and the crude product purified by flash chromatography using a stepwise gradient starting from; i) EtOAc, ii) EtOAc/Acetone/H_2O (100:100:1) and iii) EtOAc/Acetone/H_2O (10:10:1) as eluent to give 6,6'-TBDPS-Sucrose (**5**) as a white solid (4.66g, 65%). R_f = 0.40 (EtOAc/Acetone/H_2O (100:100:1)). MALDI-TOF MS (DHB+Na): Chemical Formula: $C_{44}H_{57}NaO_{11}Si_2$, calculated mass; 841.08; found: 841.81 ($M+Na^+$). **6,6'-TBDPS-isobutyric-sucrose** (**6**). 6,6'-TBDPS-Sucrose (**5**) (3.00g, 3.66mmol) was dissolved in dry pyridine (45.0mL) under N_2-atmosphere. To this solution

isobutyric anhydride (15.00mL, 90.4mmol) was added and the mixture stirred at rt overnight. Additional isobutyric anhydride (5.0mL, 15.06mmol) and a catalytic amount of 4-dimethylaminopyridine (DMAP) (50mg, 0.41mmol) were added and the mixture heated to 70°C for 6h. The solvent was removed *in vacuo* and the crude product purified by flash chromatography using hexane:EtOAc (5:1) as eluent to give 6,6'-TBDPS-isobutyric-Sucrose (**6**) as clear viscous oil (4.54g, quantitative). R_f = 0.48 (hexane:EtOAc (5:1). MALDI-TOF MS (DHB+Na): Chemical Formula: $C_{68}H_{94}NaO_{17}Si_2$, calculated mass; 1262.62; found: 1262.22 ($M+Na^+$). **6,6'-OH-isobutyric-Sucrose** (**7**) 6,6'-TBDPS-isobutyric-Sucrose (6) (4.54g, 3.66mmol) was dissolved in dry THF (20.0mL) under N_2-atmosphere. To this solution tetrabutylammonium fluoride (TBAF) in THF (1.0M, 14.65 mL, 14.65mmol) was added and the mixture stirred at rt for 4h. The solvent was removed *in vacuo* and the crude product purified by flash chromatography using hexane:EtOAc (3:1) as eluent to give 6,6'-OH-isobutyric-Sucrose (**7**) as clear viscous oil (1.78g, 64%). R_f = 0.21 (hexane:EtOAc (3:1). ^{1}H-NMR (300MHz, DMSO-d_6): δ 5.75 (d, J = 6.1 Hz, 1H), 5.50 (d, J = 3.6 Hz, 1H), 5.40 (d, J = 7.7 Hz, 1H), 5.31 (t, J = 7.4 Hz, 1H), 5.18 (t, J = 9.8 Hz, 1H), 4.87 (t, J = 5.5 Hz, 1H), 4.70 (dd, J = 10.4, 3.7 Hz, 1H), 4.29 (d, J = 11.9 Hz, 1H), 4.11 (dd, J = 12.0, 5.5 Hz, 1H), 3.69–3.44 (m, 4H), 2.64–2.49 (m, 6H), 1.13–0.96 (m, 36H). MALDI-TOF MS (DHB+Na): Chemical Formula: $C_{36}H_{58}NaO_{17}$, calculated mass; 785.83; found: 785.82 ($M+Na^+$).

6,6'-(2,4,6-triiodophenoxy)acetoxy-isobutyric-sucrose (**8**) (Fig. S1, scheme 4).

6,6'-OH-isobutyric-Sucrose (**7**) (800mg, 1.05mmol) was dissolved in dry DMF (10.0mL) under N_2-atmosphere. To this solution a pre-mixed mixture of 2-(2,4,6-triiodophenoxy)acetic acid (**3**) (1.67g, 3.15mmol), EDC·HCl (622mg, 3.15mmol) and DMAP (769mg, 6.29mmol) in dry DMF (10.0mL) were added and the reaction stirred at rt overnight. The solvent was removed *in vacuo* and the remaining yellow oil re-dissolved in CH_2Cl_2 (40mL) and washed with MQ-H_2O (3×40mL). Organic phase was dried with $MgSO_4$, filtrated and reduced *in vacou* to give light yellow oil. Final purification was achieved by flash chromatography using hexane:EtOAc (5:1) as eluent to give 6,6'-(2,4,6-triiodophenoxy)acetoxy-isobutyric-sucrose (**8**) as white foamy solid (1.56g, 83%). R_f = 0.31 (hexane:EtOAc (5:1). ^{1}H-NMR (300MHz, MeOD): δ 8.05 (s, 2H), 8.04 (s, 2H), 5.68 (d, J = 3.7 Hz, 1H), 5.56 (d, J = 7.3 Hz, 1H), 5.54 – 5.48 (m, 1H), 5.43 (t, J = 7.2 Hz, 1H), 5.37 (t, J = 9.8 Hz, 1H), 5.03 (dd, J = 10.2, 3.7 Hz, 1H), 4.70–4.06 (m, 12H), 2.73–2.45 (m, 6H), 1.36–1.04 (m, 36H). MALDI-TOF MS (DHB+Na): Chemical Formula: $C_{52}H_{64}I_6NaO_{21}$, calculated mass; 1809.47; found: 1809.59 ($M+Na^+$).

In order to obtain sufficient radiopacity of the palpable marker for visualization using real-time 2D fluoroscopy during marker placement the electron dense sucrose derivate xSAIB (**8**) containing six iodine atoms per molecules were synthesized over four steps starting from sucrose as illustrated in figure S1.

The 6,6'-hydroxyl groups of sucrose (**4**) was protected using TBDPS-Cl resulting in 6,6'-TBDPS-sucrose (**5**) as previously described by Andrade *et al.* [29]. The remaining hydroxyl groups of **5** was acylated using excess of isobutyric anhydride resulting in the formation of 6,6'-TBDPS-isobutyric-Sucrose (**6**). Both TBDPS protection groups were subsequently removed using TBAF and the 6,6'-hydroxyl groups of 6,6'-OH-isobutyric-Sucrose (**7**) acylated with 2-(2,4,6-triiodophenoxy)acetic acid (**3**) using EDC·HCl and DMAP resulting in xSAIB (**8**) in an overall yield of 35%.

Synthesis of 6'-(cyanine 7.5)-isobutyric sucrose (Sucrose septaisobutyrate Cyanine 7.5, Cy7.5-SSIB) (Fig. S1, scheme 4).

In the current synthesis, the NIR dye Cyanine 7.5 is chemically linked to the hydrophobic carbohydrate ester sucrose septaisobutyrate yielding the product Cy7.5-SSIB.

General experimental conditions: All reactions were carried out under inert atmosphere (N_2). Water sensitive liquids and solutions were transferred via syringe. Water used for washing of the isolated products was in all cases MilliQ water. Organic solutions were concentrated by rotary evaporation at 30-60°C at 200-0 mbar. Thin layer chromatography (TLC) was carried out using aluminum sheets pre-coated with silica 60F (Merck 5554). The TLC plates were inspected under UV light or developed using a cerium ammonium sulphate solution (1% cerium (IV) sulphate and 2.5% hexa-ammonium molybdate in a 10% sulfuric acid solution).

Reagents: Cyanine 7.5 NHS ester was purchased from Lumiprobe (Lumiprobe, Hannover, Germany), and dry solvents were purchased from Acros Organics (AcroSeal, extra dry over molecular sieves) (Thermo Fisher Scientific, Geel, Belgium). All other chemicals were purchased from Sigma Aldrich and were used as received.

Instrumentation: Nuclear Magnetic Resonance (NMR) of intermediates was acquired on a Bruker Ascend (Bruker, Billerica, MA., USA) 400 MHz - operating at 401.3 MHz for ^{1}H and 100.62 MHz for ^{13}C - with a 5 mm H – Broadband Dual Channel z-gradient Prodigy cryoprobe at 298 K, using the residual non-deutorated solvent residue in the NMR solvents as internal standard. NMR of the final product was acquired with an 800 MHz Bruker Avance IIIHD spectrometer equipped with a TCI cryoprobe (Bruker, Billerica, MA., USA) in order to obtain optimal spectral resolution. All coupling constants (*J*) are expressed in Hz. The FID files were processed in Mnova Suite. MALDI-TOF MS was acquired on a Bruker Autoflex Speed mass spectrometer. The matrix used for MALDI-TOF was a mixture of 2,5 dihydroxy benzoic acid (DHB) spiked with sodium trifluoroacetate in ethanol (60 mg/mL). UPLC was conducted on a Waters Acquity Ultra performance LC system with Binary solvent manager and TUV detector. Preparative HPLC was conducted on a Waters 600 pump and controller with a Waters 2489 UV/Vis detector (Waters, Milford, MA., USA).

6'-TBDPS-isobutyric sucrose (2)

Sucrose (1) (2.5g, 7.3mmol) was suspended in a solvent mix of 35 mL dry pyridine and 10 mL dry DMF. Hereafter, DMAP (0.36 g, 2.92 mmol) was added and the mixture was stirred until properly dissolved. Then, TBDPS-Cl (1.1 mL, 4.0 mmol (0.55 eq)) was added dropwise through a syringe over 10-15 minutes, and the reaction was continued overnight. 16 hours later, another portion of TBDPS-Cl (1.1 mL, 4.0 mmol (0.55 eq)) was added, after which the reaction was again left to stir overnight. Hereafter, UPLC (column: C8. Injection volume: 5 µL. Eluent: A: 0.1% formic acid in water. B: Acetonitrile, 0.1% formic acid. Gradient: 5-100% B within 6 minutes. Wavelengths 220 and 280 nm) showed conversion to mono and di-tbdps sucrose in a 2:1 relationship (retention times: 3.1 and 5.2 minutes respectively). The formed 6'-TBDPS-sucrose was not isolated, instead the mixture was reacted directly with isobutyric anhydride and then purified to give (2). Isobutyric anhydride (34 mL, 0.21 mol) was added, and the reaction was stirred at room temperature for 1 day. The reaction was followed by MALDI-TOF MS. At the point of completion, the reaction mixture was concentrated on celite *in vacuo*. Purification was done by flash chromatography (EtOAc in hexane with 2% increments). Yield = 14.8 g (50%). ^{1}H-NMR (400MHz, DMSO-D6): ^{1}H NMR (400 MHz, DMSO-d_6) δ 7.63 – 7.57 (m, 4H), 7.47 – 7.37 (m, 6H), 5.62 (d, *J* = 3.6 Hz, 1H), 5.51 – 5.46 (m, 2H), 5.39 – 5.33 (m, 1H), 5.04 (t, *J* = 9.8 Hz, 1H), 4.87 (dd, *J* = 10.4, 3.7 Hz, 1H), 4.25 (ddd, *J* = 10.3, 4.2, 2.0 Hz, 1H), 4.20 – 4.03 (m, 4H), 3.91 (dd, *J* = 12.5, 2.0 Hz, 1H),

3.87 – 3.77 (m, 2H), 2.60 – 2.51 (m, 3H), 2.47 – 2.33 (m, 3H), 1.15 – 1.07 (m, 12H), 1.07 – 0.94 (m, 40H).

^{13}C-NMR (101 MHz, DMSO-d_6): δ 175.6, 175.2 (2C), 175.0, 174.9 (2C), 174.5, 135.0 (4 C), 132.4, 132.2, 129.9 (2C), 127.8 (4C), 102.4, 89.1, 80.0, 75.2, 73.5, 69.2 69.1, 68.1, 67.1, 63.8, 63.3, 61.2 (carbohydrate carbons), 33.2 (3C), 33.1 (2C), 33.0 (2C) (CH isobutyrate), 26.4 (4C), 18.7, 18.6 (3C), 18.5 (5C), 18.4 (2C), 18.3 (2C), 18.2.

MALDI-TOF MS: Calc [M+ Na]$^+$: 1093.53. Found:1093.31.

6'-OH-isobutyric sucrose (Sucrose septaisobutyrate) (3)

6'-TBDPS-isobutyric sucrose (2) (14.8 g, 13.8 mmol) was dissolved in dry THF (80mL). Acetic acid (12 mL, 0.21 mol) was carefully added dropwise. The reaction mixture was then cooled down, and 1.0M TBAF solution in THF (83 mL, 83 mmol) was added over 10-15 minutes through a syringe. The reaction was allowed to heat to room temperature over 30 minutes, hereafter it was warmed to 40^{0}C and stirred at this temperature overnight. Then, TLC (Hexane:Ethyl acetate 3:1) showed completion of the reaction (rf product: 0.2). The reaction mixture was cooled to room temperature and first hexane (300 mL) then demineralized water (300 mL) was added. The mixture was stirred for 10 minutes, and hereafter poured into a separatory funnel. The organic phase was collected and the water phase was extracted with hexane (2 × 300 mL). The combined organic phases were washed with HCl (aq) (500 mL, pH= 2) and subsequently with phosphate buffer (3 × 300 mL, pH = 6.8). The organic phase was concentrated on celite and then purified by dry column purification (EtOAc in hexane with 2-4 % increments) to give the product. Yield 5.1 g (89.5 %). Texture: transparent oil. ^{1}H-NMR (400 MHz, Chloroform-d): δ 5.68 – 5.39 (m, 4H), 5.18 (t, J = 10.4 Hz, 1H), 4.94 (dd, J = 10.4, 3.6 Hz, 1H), 4.36 – 4.14 (m, 3H), 4.10 – 3.93 (m, 3H), 3.84 (dd, J = 12.9, 2.8 Hz, 1H), 3.60 (dd, J = 12.9, 3.6 Hz, 1H), 2.69 – 2.24 (m, 7H), 1.34 – 0.99 (m, 42H).

^{13}C-NMR (101 MHz, Chloroform-d): δ 176.8, 176.3 (2C), 176.1, 176.0, 175.9, 175.2, 102.8, 90.2, 81.4, 75.6, 72.5, 70.0, 69.5, 69.1, 67.2, 64.0, 60.8, 60.7, 34.0 (4C), 33.9 (2C), 33.8, 19.2, 19.1, 19.0 (4C), 18.9 (5C), 18.8 (2C), 18.5.

MALDI-TOF MS: Calc [M+ Na]$^+$: 855.41. Found: 855.20.

6'-(Cyanine 7.5)-isobutyric sucrose (Sucrose septaisobutyrate Cy 7.5) (4)

6'-OH-isobutyric sucrose (3) (14 mg, 0.017 mmol) was dissolved in dry DCM (3 mL). Then, Cyanine 7.5 NHS ester (15 mg, 0.019 mmol) was added, followed by addition of triethylamine (10 µL, 0.072 mmol). The reaction was stirred at room temperature for 2 days. Then, TLC (Hexane:Ethyl acetate 3:1) showed full conversion. The organic phase was concentrated *in vacuo*, the compound redissolved in methanol (2 mL) and purified by preparative HPLC (Column: Xterra C8. Eluent system: A: 0.1% TFA in water. B: Acetonitrile, 0.1% TFA. Gradient: 75-100% B within 15 minutes). Yield: 15.2 mg (62 %). Texture: green powder. ^{1}H-NMR (800 MHz, DMSO-d_6): δ 8.24 (dd, *J* = 11.5, 8.5 Hz, 2H), 8.09 – 8.02 (m, 4H), 7.84 – 7.63 (m, 6H), 7.53 – 7.47 (m, 2H), 6.19 (dd, *J* = 26.1, 14.0 Hz, 2H), 5.62 (d, *J* = 3.6 Hz, 1H), 5.50 (d, *J* = 7.4 Hz, 1H), 5.40 – 5.31 (m, 2H), 5.10 – 5.06 (m, 1H), 4.93 – 4.85 (m, 2H), 4.33 – 4.01 (m, 8H), 3.86 (t, *J* = 6.8 Hz, 1H), 3.76 – 3.74 (m, 2H), 2.93 (q, *J* = 6.7 Hz, 1H), 2.59 – 2.53 (m, 10H), 2.47 – 2.30 (m, 3H), 2.07 (s, 3H), 1.94 (br s, 6H), 1.89 – 1.85 (m, 2H), 1.80 – 1.74 (m, 2H), 1.66 – 1.34 (m, 8H), 1.27 – 0.83 (m, 42H).

MALDI-TOF MS: Calc [M+ H]$^+$: 1464.78. Found: 1464.73.

Cryo-SEM imaging of X-mark and XPV-mark

Cryo SEM of hydrated X-mark and XPV-mark formulations revealed an internal pore structure in each gel with pores of different sizes. Pore size decreased in diameter the further from the surface of the gel the pore was located. Larger pores were often surrounded by much smaller pores in both formulations. The XPV-mark formulation exhibited pores that were consistently larger than those observed in the X-mark formulation as shown in Supplementary figure S2 where the XPV-mark formulation had pores up to ~250 µm in diameter whereas the X-mark formulation only had pores with a diameter up to ~125 µm.

Materials and methods: X-mark and XPV-mark formulations were injected into buffer in a 12 well plate and aged for 1 week at 37 ºC to ensure adequate ethanol diffusion out of the gel. Gel samples were then adhered to SEM stubs with a 50:50 mixture of colloidal graphite powder (agar scientific) and Tissue-Tek OCT compound (Ted Pella) and plunge frozen in liquid nitrogen. Frozen samples were then loaded onto a Leica EM VCT 100 Cryo Transfer Shuttle and transferred to a Leica EM MED020 freeze fracture and high vacuum coating system. Samples were then fractured, sublimated for 1 minute at -90ºC and sputter coated with 6 nm of carbon/platinum. After coating, the samples were transferred via the VCT 100 Cryo Transfer Shuttle under vacuum and at -140ºC to the Thermo Scientific Quanta 3D FEG FIB/SEM for subsequent SEM imaging. Imaging was performed at high vacuum at -140ºC with an accelerating voltage of 2 kV.

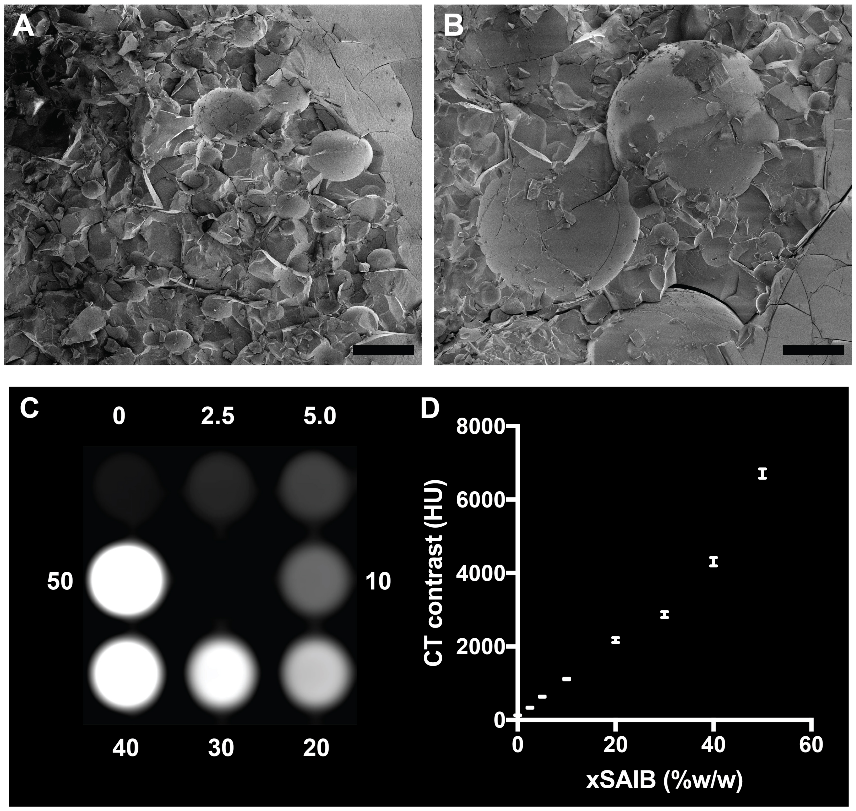

Fig. S2. Cryo-SEM images of hydrated X-mark and XPV-mark and CT contrast as function of xSAIB content in XPV-mark. hydrated X-mark (**A**) hydrated XPV-mark (**B**) (scalebars: 100 µm). XPV-mark formulations containing 0, 2.5, 5.0, 10, 20, 30, 40 and 50 %w/w xSAIB were CT imaged (**C**) and the corresponding CT contrast was quantified and presented as function of the xSAIB content (**D**).

X-ray Visibility of XPV-mark - CT contrast dependency on xSAIB content

Liquid marker formulations with 0, 2.5, 5.0, 10, 20, 30 ,40 or 50w/w% xSAIB in LOIB and fixed amount of EtOH (18.0 w/w%) and DC2 (0.10 w/w%) were prepared using the general procedure. The liquid marker formulations (200 μL) were added to separate wells in a 96 well microtiter plate. Evaluation of radiopacity of the liquid marker formulations as a function of xSAIB concentration was performed on a Siemens SOMATOM Definition AS^+ CT scanner (Siemens Health Care, Erlangen, Germany). The CT settings were a tube voltage of 120 kVp, 300mAs, 200mm FOV and extended CT-scale. Slices were reconstructed in a coronal plane with a B30s reconstruction kernel. HU contrast was quantified using Eclipse v 13.7 software package. The CT contrast was shown to depend linearly on the xSAIB content up to 40% w/w for XPV-mark formulations, where after the CT contrast increased further non-linearly. The CT contrast followed the regression line:

$$CT(HU) = 100 \cdot C_{xSAIB}\left(\% \frac{w}{w}\right) + 100, \quad (R^2 = 0.992)$$

X-mark stability – effect of pH

Markers composed of LOIB:xSAIB:EtOH:DC2 (51.9:30.0:18.0:0.1) (300 μL, n = 6 × 3) were formed by injection into separate glass vials containing sterile filtrated MilliQ-H_2O (10.0 mL) using a high-precision Hamilton syringe. Markers were stored at 37°C for 24h to allow ethanol efflux from the markers into the aqueous solution. After 24h, markers were randomly distributed into 6 groups (6 × 3); one control group and five groups individually incubated at pH 4, 5, 6, 7 and 8, respectively, using pH adjusted sterile filtrated BIS-TRIS propane (pKa: 6.8 and 9.0) and Citric acid (pKa: 3.1, 4.8 and 6.4) buffer for 30 days at 37°C.

Analytical HPLC analysis was conducted in order to detect the formation of TIPA caused by chemical hydrolysis of xSAIB in the X-mark markers in the degradation buffer medium as illustrated in Fig. S3. TIPA was chosen as the preferred analyte to monitor over time due to its UV-absorbance at 256nm.

200 μL aliquots from the aqueous buffer phase above all markers were removed at designated intervals after 10, 20 and 30 days and replaced with fresh buffer to mimic the sink effect *in vivo*. After 30 days, aliquots and markers dissolved in MeCN were analyzed by analytical HPLC to monitor the pH dependent degradation of the markers based on the AUC from 2-(2,4,6-triiodophenoxy)acetic acid (TIPA) formed by hydrolysis of xSAIB.

A

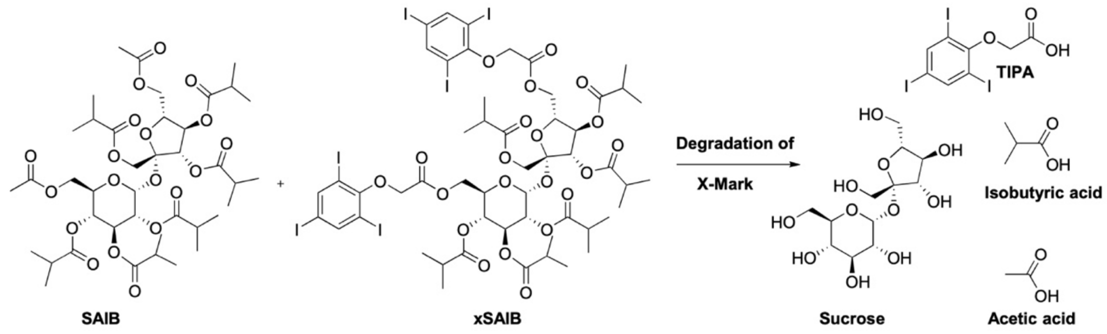

B

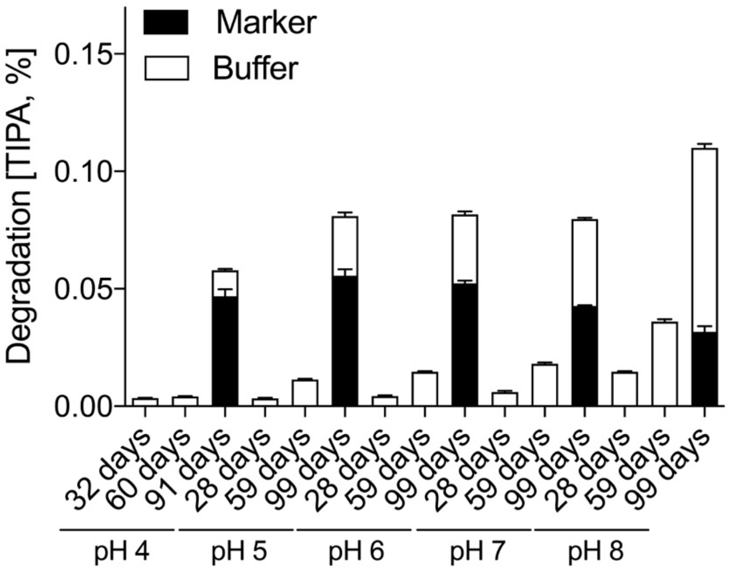

Fig. S3. X-mark components after complete efflux of EtOH and degradation (% of xSAIB) of X-mark. SAIB and xSAIB and the primary degradation products formed by chemical hydrolysis of the multiply ester bonds of SAIB and xSAIB (**A**). Markers in buffer at pH 4.00-8.00 inside glass vials at Day 0, ~Day 30, ~Day 60 and ~Day 90 following incubation at 37°C. Reported values represents mean ± SEM, n = 3 (**B**).

Analytical HPLC analysis was conducted using a Shimadzu LC-2010 analytical HPLC (Shimadzu Corp., Kyoto, Japan) by employing a Waters XTerra® C_8 5 µm (4.6 x 150 mm) column (Waters Corporation, MA, USA). Analysis was conducted using a linear gradient from 0-100% Eluent B over 15min followed by 100% B for 5 minutes (Eluent A: 5% MeCN in MQ-H_2O + 0.1% TFA; Eluent B: MeCN + 0.1% TFA). Flow rate: 1.00mL/minutes, injection volume: 25µL and UV-detection at 220 nm and 256 nm. Minimal X-mark marker degradation (< 0.15%) was observed after incubation of markers at pH 4.00, 5.00, 6.00, 7.00 and 8.00 at 37°C for >90 days as monitored by the amount of TIPA released from the markers. The pH of the degradation medium had little effect on the hydrolysis rate of the markers and was found to affect the partition of TIPA between the hydrophobic markers and the hydrophilic buffer phase.

Serum cytokine levels and tolerability of X-mark in mice over a 98-day study period

Please refer to main text materials and methods section for study details. Blood sampling was performed by facial vein puncture and direct collection of blood into Eppendorf tubes. The blood samples are allowed to reach room temperature. The blood samples are subsequently centrifuged (10 minutes, 4°C, 1500 rpm.) and the serum collected and stored at -80°C until further processing.

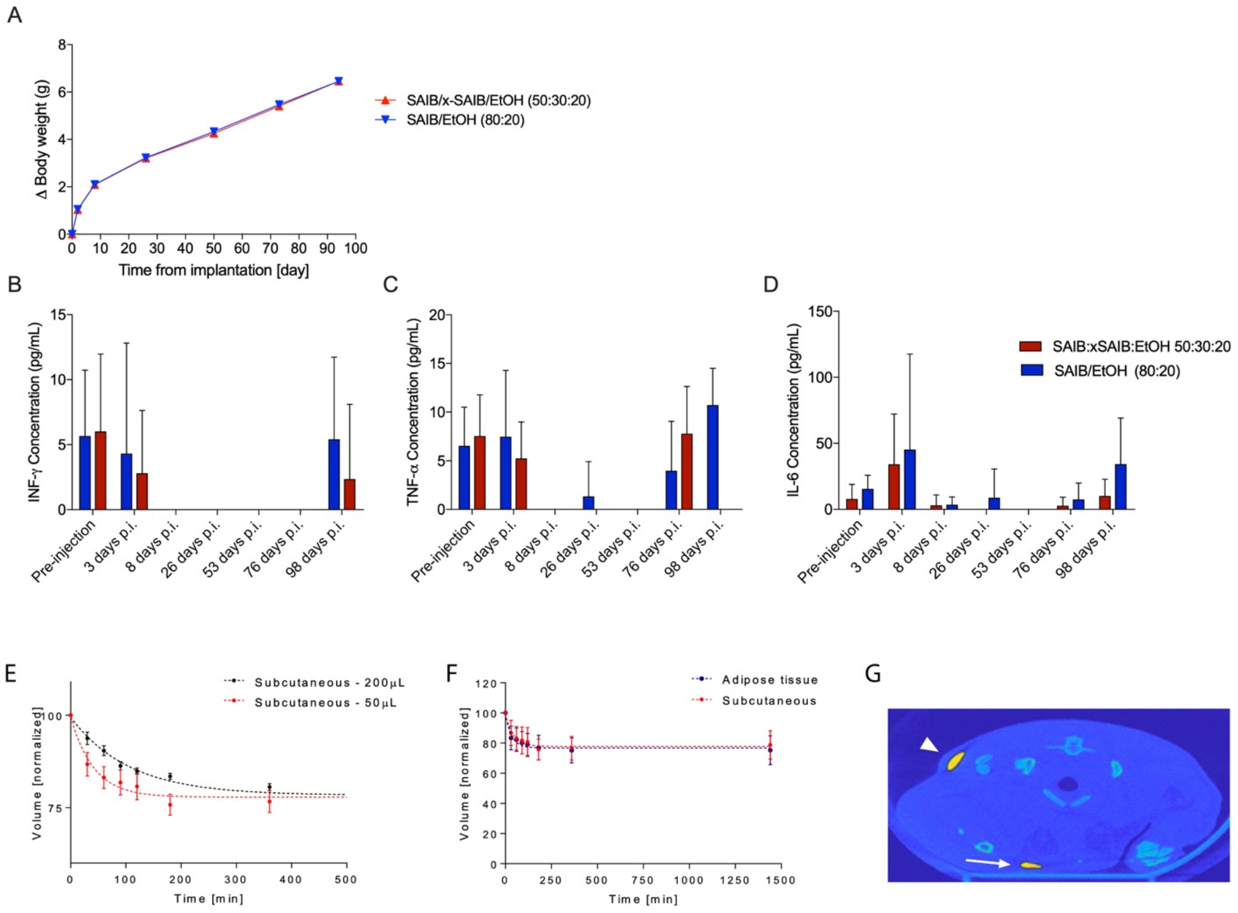

Fig. S4. Tolerability of X-mark in mice and solvent efflux kinetics in rats as a function of injection volume and tissue composition. (**A**) Body weight expressed as mean weight gain of SAIB:xSAIB:EtOH (50:30:20) and SAIB:EtOH (80:20) groups (n=8) over the 98-day study period. (**B,C,D**) Blood samples were collected prior to injection of SAIB:xSAIB:EtOH (50:30:20) or SAIB:EtOH (80:20) and again 3d, 8d, 26d, 53d, 76d and 98d from both groups for assessment of cytokine response. Serum levels of IFN-γ (**B**), TNF-α (**C**) and interleukin 6 (IL-6) (**D**) were analyzed using a bead-based sandwich immunoassay and a commercially available Mouse Cytokine/Chemokine Magnetic Bead Panel kit (Cat#MCYTOMAG-70K-03) on the Luminex LX100 (Millipore Corporation, Burlington, MA., USA). (**E**) EtOH efflux from X-mark (SAIB:xSAIB:EtOH (50:30:20)) injections of 50 μl and 200 μl, respectively, evaluated by reduction in segmented volume of the injected markers on CT scans performed at multiple time points (0, 30, 60, 90, 120 minutes and 3, 6 and 24 hours) up to 24 hours after injection. Comparison by t-test demonstrated a significant difference in mean time to complete ethanol efflux between subcutaneous 50 μL markers (165 ± 45 minutes) and 200 μL markers (330 ± 133 minutes) (p=0.009). (**F**) EtOH efflux from 50 μL X-mark (SAIB:xSAIB:EtOH, 50:30:20) in adipose tissue and sub cutaneous space, respectively, evaluated by reduction in segmented volume of the injected markers on CT scan performed at multiple time points (0, 30, 60, 90, 120 minutes and 3, 6 and 24

hours) up to 24 hours after injection. Comparison by t-test demonstrated no significant difference in mean time to complete ethanol efflux between subcutaneous markers (165 ± 45 minutes) and markers in adipose tissue (138 ± 45 minutes) ($p = 0.675$). (**G**) Representative slice of 50 μL X-mark (SAIB:xSAIB:EtOH, 50:30:20) in adipose tissue (arrow) and sub cutaneous space (arrow head) of rat on 24 hours pi. CT-scan. The black line surrounding the markers, highlighted in yellow, is the marker contouring delineation used for image analysis.

EtOH solvent efflux of X-mark

SAIB:xSAIB:EtOH (50:30:20) ethanol efflux kinetics as function of injection volume and injected tissue composition during the initial 24 hours after injection.

Eight-week-old female Wistar rats were injected subcutaneously (50 μL (n=7) and 200 μL (n=6)) and in the subcutaneous caudal fat pads (50 μL (n=7)) with X-mark gel (SAIB:xSAIB:EtOH (50:30:20)) (25mm 25 G. needle). Micro-CT scans (50 kVp, 520 μA, 480/steps, 300 ms/exposure time, 0.092×0.092×0.092 mm/voxel) were performed 0, 30, 60, 90, 120 minutes and 3, 6 and 24 hours after injection using a small-animal SPECT/CT scanner (NanoScan, Mediso, Budapest, Hungary). Markers were semi-automatically segmented using the Chan-Vese segmentation model, and the time resolved marker volume reduction for both injection sites (subcutaneous vs. adipose tissue) and for the two volumes (50 μL vs. 200 μL) were determined. The segmented markers were analyzed in terms of; 1) marker volume vs. time and 2) marker surface area 24 hours post injection. The ethanol efflux kinetics from X-mark were fitted using an exponential function:

$$V_{X\text{-}mark}(t) = (V_{X\text{-}mark,start} - V_{plateau}) \cdot \exp(-K \cdot t) + V_{plateau}$$

Where $V_{X\text{-}mark(t)}$ and $V_{X\text{-}mark,start}$ are the X-mark volume at time t and at start (initial volume). $V_{plateau}$ is the final volume after complete efflux occurred, and K is the exponential decay constant. 95% complete ethanol efflux was defined as: $(V_{x\text{-}mark(t)} - V_{plateau}) / (V_{X\text{-}mark,start} - V_{plateau}) = 0.05$, corresponding to 5% volume change remain to occur. The time required for 95% completion of the efflux was calculated according to: $T_{95\%} = \ln(20)/K$.

Practicability and performance of X-mark in pigs

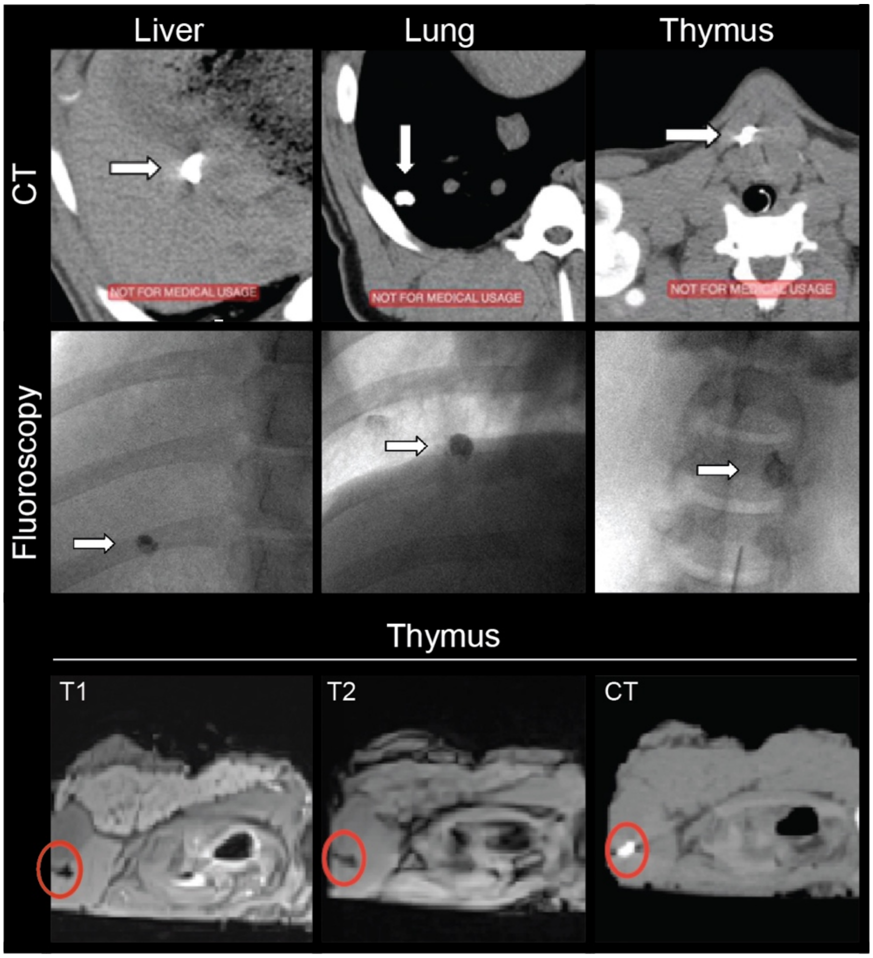

Fig. S5. Evaluation of injection practicability and short-term performance of X-mark in pigs. Practicability and imaging characteristics were investigated by injection of SAIB:xSAIB:EtOH (50:30:20) into liver using EUS (22G. endoscopic injection needle), into lung by percutaneous fluoroscopy guided injection (22G. 63 mm needle) and thymus using EBUS (22G. endoscopic injection needle). Imaging performance was evaluated by CT imaging (top row) and fluoroscopy (middle row) and the marker in thymus was imaged post mortem in one pig imaged using MRI and CT (bottom row, MRI T1 and T2 weighted images and CT).

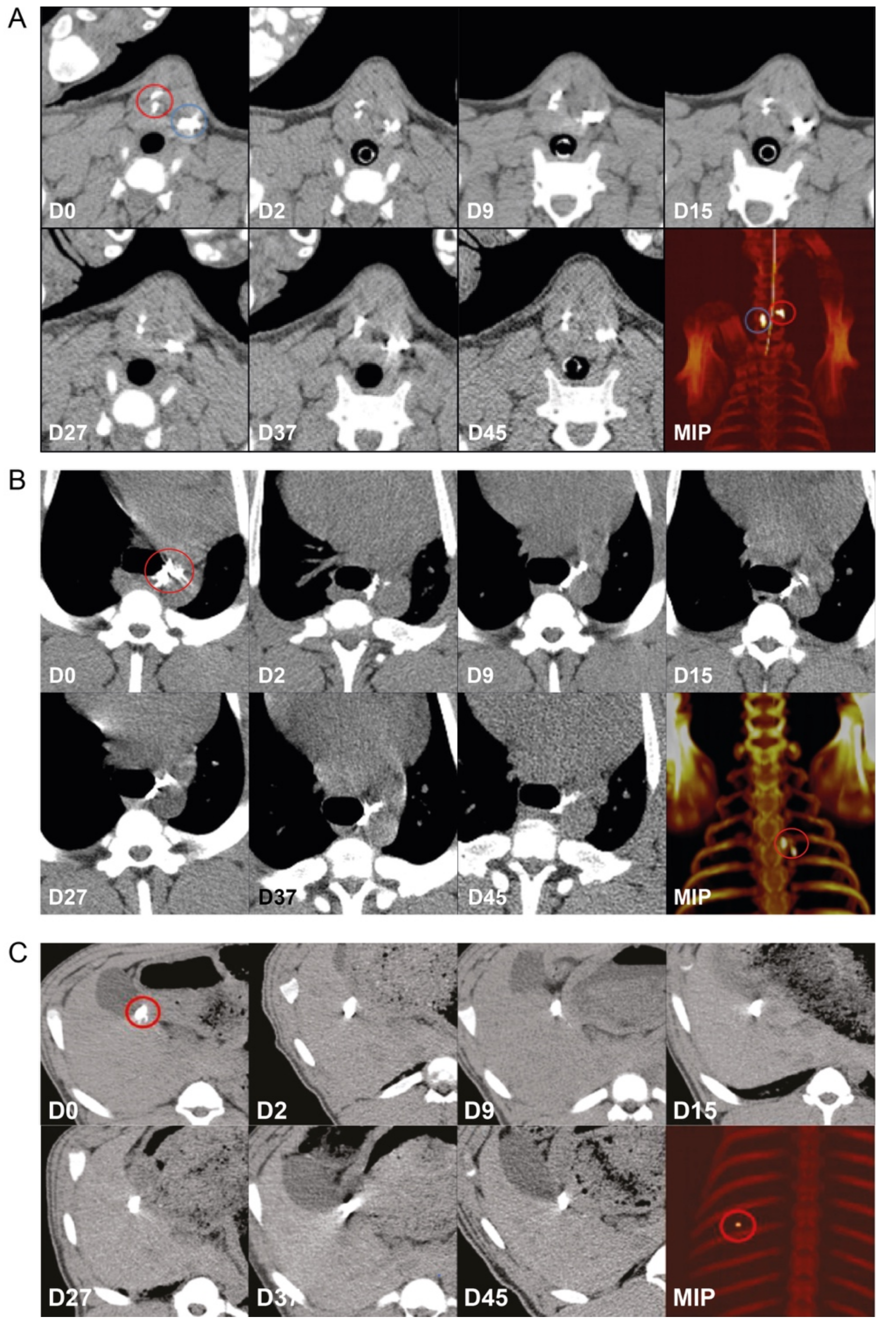

Fig. S6. Six-week tolerability and performance of X-mark in pigs across clinical imaging modalities. Characteristics and imaging properties were investigated by injection of across a range of SAIB:xSAIB:EtOH (50:30:20) into various locations, using EBUS, EUS and percutaneous injection technique, in experimental pigs. Injections included (**A**) thymus, (**B**) mediastinal lymph node, (**C**) liver. For the CT-evaluation all measurements of HU and contouring were performed in Matlab 2013a or Osirix. A maximum intensity projection (MIP) was used for three directions to select the liquid marker for evaluation. Three-dimensional ROIs of the liquid marker was then used for evaluation with lower window setting of 400 HU. (**A**) EBUS assisted injection of two 200 µl marker depots using a 22G. endoscopic injection needle. Please refer to video S1 for recorded EBUS assisted injection of marker (**B**) EUS assisted injection of one 200 µl marker depot using a 22G. endoscopic injection needle. (**C**) Ultrasound guided percutaneous injection of one 200 µl marker depot using a 22G. 63 mm needle. Images from injection sites includes Day 0, 2, 9, 15, 27, 37, 45, CT scans and the maximum intensity projection (MIP) from CT scan day 2.

Practicability and performance of XPV-mark in pigs

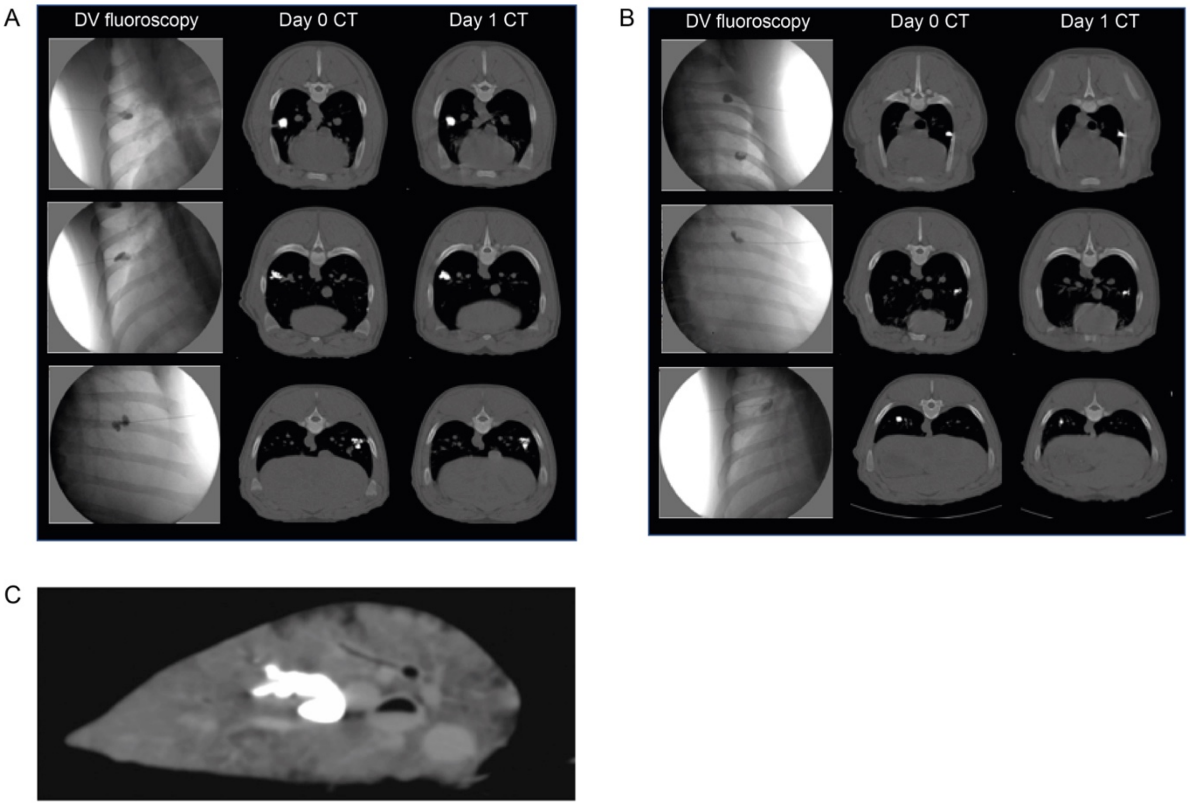

Fig. S7. Performance of XVP-mark (LOIB:xSAIB:EtOH:DC2 (69.5:20:10:0.25)) injected percutaneously into multiple locations in pig lungs. Pigs (~45 kg) were injected day 0 under fluoroscopy guidance and CT scans were performed directly after injection and again approximately 24 hours later (**A** and **B**). Injection volumes were either 600µl (**A**) or 300µl (**B**). Please refer to video S2 for real time fluoroscopy of injection of 600µl XPV-mark (DV: dorsoventral). (**C**) CT scanning of lungs after removal from the thoracic cavity allow for the determination of the center of marker to lung-surface distance at which marker was palpable, maximal center of marker to lung-surface distance was estimated to 17 mm.

Spectroscopic characterization of the Cy7.5-SSIB dye

The NIR dye Cy7.5-SSIB was formulated in markers based on SAIB or LOIB and characterized by fluorescence or absorbance. The fluorescence emission was furthermore investigated as function of dye concentration in SAIB based markers.

The marker formulations: SAIB:xSAIB:EtOH and LOIB:xSAIB:EtOH:DC2 containing Cy7.5-SSIB were prepared by simple mixing as described above.

Fluorescence spectrum: For SAIB:xSAIB:EtOH Cy7.5-SSIB formulations, 1 mL of samples were pipetted into quartz cuvettes (Helma 10.00 mm), and the fluorescence emission from 780 nm to 900 nm was recorded using a fluorescence spectrometer (OLIS SLM8000, USA). The emission spectra were recorded using an excitation wavelength of 768 nm, scan-time of 45s, and a slit-width of 8 nm. For the LOIB:xSAIB:EtOH:Cy7.5-SSIB formulations, 1 mL of samples were pipetted into quartz cuvettes (Helma 10.00 mm), and the fluorescence emission from 800 nm to 1100 nm was recorded using a fluorescence spectrometer (OLIS DM45, USA) with excitation/emission bandwidth of 26 nm and integration time of 0.2 seconds. An excitation wavelength of 768 nm was employed.

The UVvis spectra of Cy7.5-SSIB marker sample was recorded using a multimode microplate reader (Tecan, Sweden), e.g. 0.2 mL of the Cy7.5-SSIB formulations 0.05 mL of the Cy7.5-SSIB formulation with DC2 were pipetted into 96-well plate, and the UV-vis spectra from 550 nm to 1000 nm was measured.

Fluorescent markers containing Cy7.5-SSIB were prepared and spectroscopically characterized. The results are presented in fig. S8. The fluorescence emission of Cy7.5-SSIB in SAIB:xSAIB:EtOH 70:10:20 displayed a dye concentration dependent change in the emission intensity and a gradual shift in peak intensity from 804 to 844. As the concentration of Cy7.5-SSIB was increased, the emission intensity increased until 0.003% w/w after which it decreased indicating dye self-quenching. UVvis analysis of LOIB:xSAIB:EtOH:DC2 displayed absorption peaks at 590 nm 803 nm corresponding to absorption from the 0.1% w/w DC2 dye and 0.01% w/w Cy7.5-SSIB dye respectively. A broad emission peak centred at 845 nm was determined from 0.01% w/w Cy7.5-SSIB in LOIB:xSAIB:EtOH:DC2.

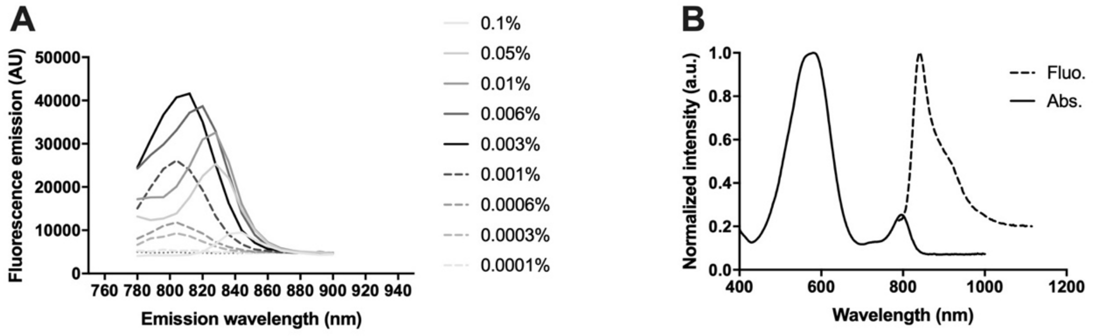

Fig. S8: Fluorescence emission of Cy7.5-SSIB when formulated in SAIB:xSAIB:EtOH 70:10:20 or LOIB:xSAIB:EtOH:DC2 70:10:20:0.1. (**A**) Fluorescence emission of Cy7.5-SSIB in SAIB:xSAIB:EtOH 70:10:20 marker formulation for different Cy7.5-SSIB dye concentrations. (**B**) Normalized absorbance and emission of LOIB:xSAIB:EtOH:DC2:Cy7.5-SSIB 70:10:20:0.1:0.01.

Practicability and performance of XPVN-mark in pigs

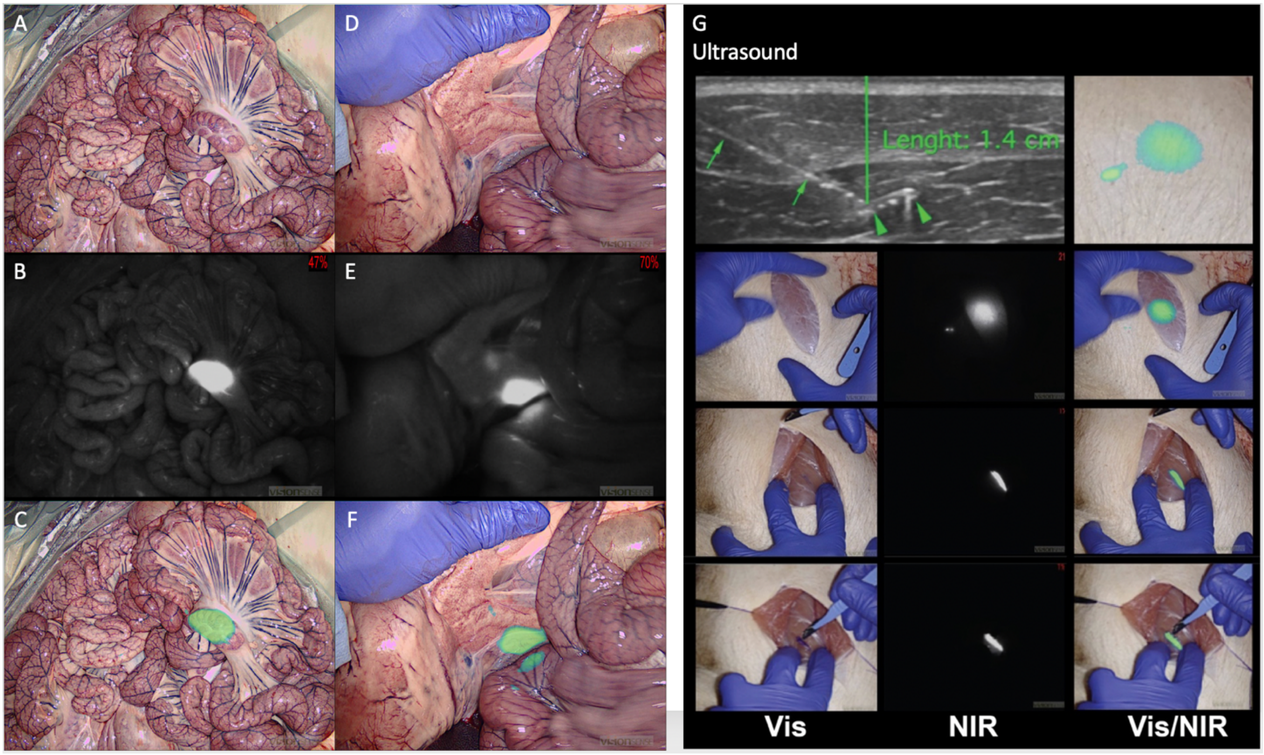

Fig. S9: NIR-guided localization of a mesenteric lymph node and region in pancreas of a pig and NIR-guided surgical localization of microchip marked with XPVN-mark. XPVN-mark was injected (23G/25mm needle) in mesenteric lymph node and in the pancreas (approximately 50 µL) of a pig. Before injection the colon and cecum sections were removed. The intestines were repositioned after injections and a blinded experienced veterinary surgeon asked to identify the injection location under visual and NIR guidance. Injected mesenteric lymph node (**A**) visual color image, (**B**) NIR and (**C**) visual color/NIR overlay. Injected region of the pancreas (**D**) visual color image, (**E**) NIR and (**F**) visual color/NIR overlay. Images were acquired using the Visionsense Iridium. To mimic the clinic situation of having to identify a specific lesion, a microchip was inserted in the medial aspect of the thigh musculature of a pig (**G**), and identified and injected by ultrasound guidance with XPVN-mark (50 µL, 21G/76 mm needle, 1 ml syringe) (top left). The location of the injected XVPN-mark was identified by NIR imaging (Iridium, Visionsense) using visual and NIR overlay (top right). The microchip was recovered surgically under visual/NIR overlay guidance. The three pictures demonstrate the information provided by visual (left row), NIR only (middle row) and visual and NIR overlay (right row). Images were acquired using the Visionsense Iridium (Photo Credit S9A-G: Jonas R. Henriksen, Technical University of Denmark).

Radiolabeling of XVPN-mark:

Radiolabeling of the marker $^{64}Cu^{2+}$: A solution of 8HQ in ethanol (200 µL, 500 µM) and pure ethanol (300 µL) was mixed with dry [^{64}Cu]$CuCl_2$ (300 MBq) and stirred at room temperature, 400 rpm, for 18 hours. Ethanol solvent was evaporated at 50°C using argon flow for 20 minutes. Then 1 mL of marker formulation (LOIB:xSAIB:EtOH:DC2:Cy7.5-SSIB 70:10:20:0.1:0.01) was added to the dry film of ^{64}Cu(8HQ) and hereafter the formulation was stirred at 50°C, 400 rpm for 2 hours. The radiolabeled marker formulation (660µL) was transferred to a new glass vial and the radioactivity was measured by dose calibrator (Comecer, VDC-505). Non-radioactive gel formulation (1.5 mL) was added to dilute the formulation to 20 MBq/mL. The final formulation was homogenized by further stirring at 50°C, 400 rpm for 20 minutes and vortexing.

Radiolabeling of the marker $^{177}Lu^{3+}$: [^{177}Lu]LuCl$_3$ in 0.05 M HCl (300 MBq) was evaporated to dryness at 50°C under a stream of Ar for about 1 h in a 2 mL HPLC vial, to which was added 8HQ in ethanol (1 mM, 100 µL). The mixture was magnetically stirred at 50°C for 1 h to form the ^{177}Lu(8HQ) complex. After that, 900 µL gel formulation composed of SAIB:GTO:xSAIB (72:18:10) was added and stirred for 2 h at 50°C.

Incorporation of ^{125}I-SSIB and *in vitro* retention of radioactivity: Approximately 3 x 100 µL of the gel solution containing the radiolabel was transferred to glass vials containing Dulbecco's phosphate buffered saline (3.0 mL). At several time points, 2 mL of the medium was removed and replaced with 2 mL fresh medium. The radioactivity left in the vial was measured after this replacement. After the last measurement (day 10), the entire medium was removed and replaced with fresh medium. The radioactivity in the removed medium aliquots was also measured. Retention of radioactivity was observed in the markers, with measured radio-activities in the gel dropping during the first day of measurement to about 95%, reaching a slowly declining plateau. In the final measurement point after 10 days of incubation, where only the radioactivity in the marker was measured, the retention was 95.5 ± 0.5% (n = 3). The initial drop in retained radioactivity could be attributed to burst release but since it was not mirrored by the radioactivity in the released medium, it is likely that the initially observed drop is a consequence of the geometric shape of the gel changing as ethanol is released and the gel settles. During the initial day of monitoring, a release of only 0.17 ± 0.08% (n = 3) was observed in the removed medium, with a slow and steady increase to about 1% released in total over the consecutive 10 days. The data demonstrate high retention of I-125 radioactivity in the LOIB:xSAIB:EtOH:DC2:Cy7.5-SSIB marker system.

XPVN-[^{64}Cu]-mark PET/CT analysis: On PET/CT images a manual large volume of interest (VOI) was constructed. This VOI was then segmented using a HU>400 cut-off to delineate the gel. The total activity of ^{64}Cu in the gel was determined from the constructed VOI and reported as total percentage of ^{64}Cu injected dose in the gel (%ID/gel). Activity of ^{64}Cu in the liver and kidney was determined by constructing a VOI in these organs based on the PET/CT scans and percentage of ^{64}Cu injected per gram reported (%ID/g) (figure S10A-C).

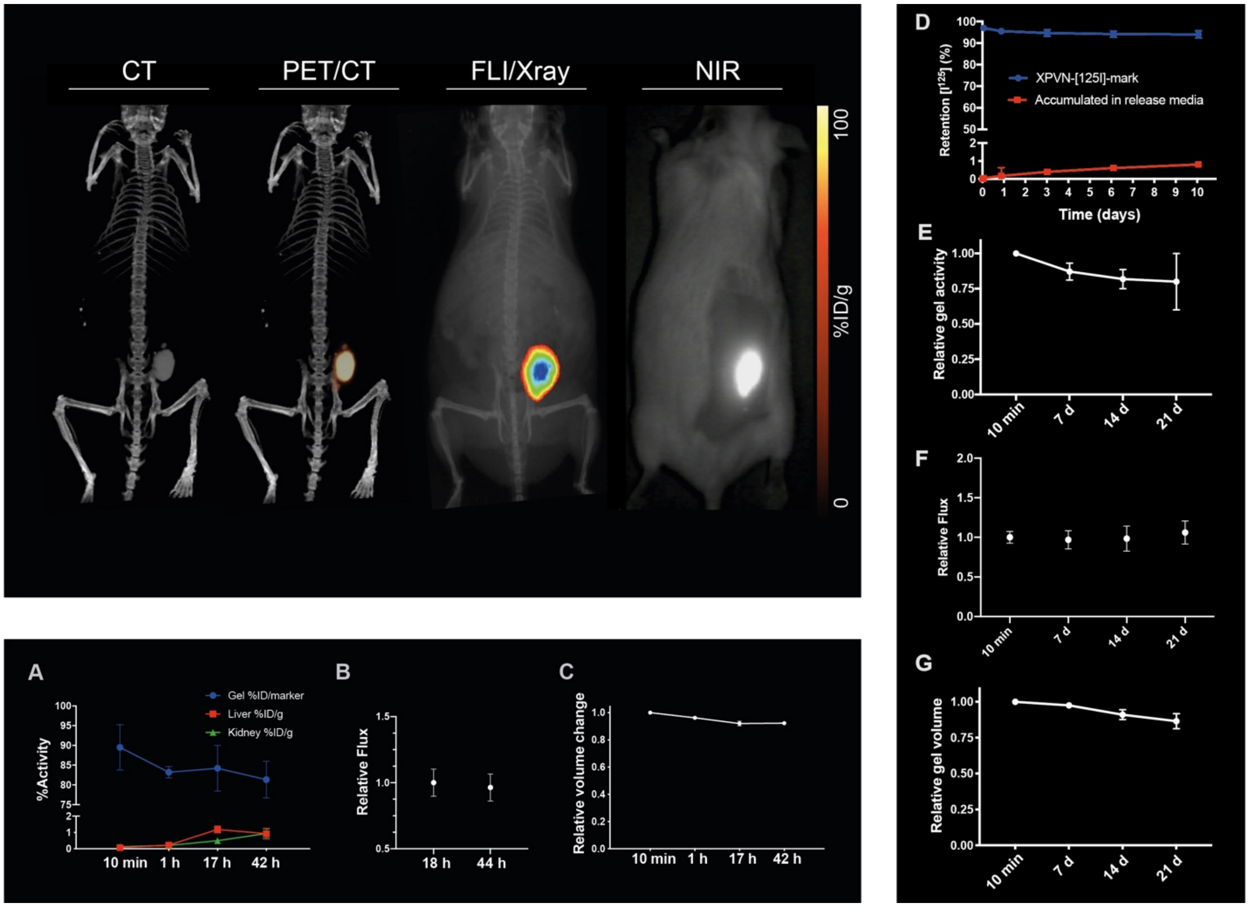

Fig. S10. Biodistribution of XPVN-[^{64}Cu]-mark and relative changes in fluorescence and gel volume over time and ^{125}I activity changes during bench testing and the three-week SPECT/CT study period of XPVN-[^{125}I]-mark. XPVN-[^{64}Cu]-mark: Mice were subcutaneously injected with ^{64}Cu(8HQ) radiolabeled LOIB:xSAIB:EtOH:DC2:Cy7.5-SSIB 70:10:20:0.1:0.01 marker and corresponding changes in ^{64}Cu biodistribution, NIR fluorescence intensity and marker volume. (**A**) ^{64}Cu biodistribution in the marker, liver and kidney as function of time. (**B**) Relative fluorescence flux from the marker after 18h and 44h post injection. (**C**) Relative volume change of the marker given as function of time post injection. (**D**) In vitro retention in gel and release to media of ^{125}I from 100 µL XPVN-[^{125}I]-mark suspended in PBS. XPVN-[^{125}I]-mark: Mice were subcutaneously injected with ^{125}I-labelled LOIB:xSAIB:EtOH:DC2:Cy7.5-SSIB marker, and corresponding changes in relative ^{125}I gel activity (**E**), relative gel fluorescence (**F**) and relative gel volume (**G**) were extracted from SPECT/CT and fluorescence scans as function of time after injection. All relative measures (volume, flux, activity) were normalized to the first timepoint.

Movie S1. Ultrasound-guided bronchoscopy injection of X-mark in a pig.

Movie S2. Fluoroscopy-guided percutaneous injection of XPV-mark in the lung of a pig.

Movie S3. Near-infrared surgical camera guided localization of XPVN-mark injected in a mesenteric lymph node of a pig.

Movie S4. Near-infrared surgical camera guided dissection of "foreign body mimic" marked by ultrasound guided injection of XPVN-mark.
